# Quantitation of Stromal Distortion Across the Full Depth of Keratoconic Corneas

**DOI:** 10.1167/iovs.67.12.58

**Published:** 2026-10-23

**Authors:** Poncia Nyembo Kasongo, Djida Ghoubay, Jean-Marc Allain, Malika Hamrani, Cristina Georgeon, Vincent Borderie, Gaël Latour, Marie-Claire Schanne-Klein

**Affiliations:** 1 Laboratoire d'Optique et Biosciences, CNRS, Inserm, École Polytechnique, Institut Polytechnique de Paris, Palaiseau, France; 2 Sorbonne Université, Hôpital National de la Vision des 15-20, INSERM, Institut de la Vision, Paris, France; 3 Laboratoire de mécanique des solides, CNRS, École polytechnique, Institut Polytechnique de Paris, Palaiseau, France; 4 Université Paris-Saclay, Gif-sur-Yvette, France

**Keywords:** corneal stroma, collagen, two photon imaging, keratoconus, corneal biomechanics

## Abstract

**Purpose:**

This study aimed at gaining new insights into the pathophysiology of keratoconus by providing a comprehensive and quantitative characterization of stromal structural alterations throughout the full thickness of keratoconic corneas and in regions both proximal to and distant from the cone.

**Methods:**

Complementary three-dimensional imaging techniques were combined in a pilot exploratory series of four keratoconic and four control human corneas: clinical spectral-domain optical coherence tomography, highly-resolved line-field confocal optical coherence tomography and second harmonic generation (SHG) microcopy. Several structural metrics were computed from SHG images using automatic image analysis pipelines.

**Results:**

Quantitative SHG imaging revealed striking structural shifts in keratoconic corneas compared to healthy controls. Most notably, we observed a significant rise in normalized striation Endpoints (*P* = 0.008) and End/Branch ratio (*P* = 0.015), consistent with the presence of wavy lamellae and the loss of the plywood-like structure found in control corneas. Importantly, these structural markers remained consistent in both the cone and outside the cone regions. Quantitative processing of collagen orientation revealed a single widely-spread orientation as opposed to two main orthogonal orientations in control corneas, and slight shifts in orientation between superimposed lamellae rather than the abrupt changes observed in control corneas.

**Conclusions:**

We provide new quantitative data on the structural alterations associated with keratoconus that could serve as useful biomarkers for earlier detection. Our findings are consistent with a decreased stromal stiffness and an increase in the corneal birefringence. They are also in favor of a global stromal disorder rather than a purely localized disorder.

Keratoconus is a structural pathology that affects the cornea. It manifests as thinning and weakening of the tissue, and can lead to a conical deformation of the tissue in the most advanced stages of the pathology.^1^^,^^2^ The vision is then strongly impaired and a corneal graft may be needed in the most severe cases. Because keratoconus affects an increasing number of people, with a global incidence rate of 1.5 to 25 cases per 1000 persons/year, it is now the third most common cause of corneal transplantation in developed countries.^2^^–^^4^ The main risk factors reported to date are mechanical stimulation (rubbing) of the eyes, genetic factors, asthma, allergies, and eczema.^2^^,^^3^ Nevertheless, the understanding of keratoconus pathogenesis and etiology is still limited, and its prevalence increase prompts a great deal of investigations.

This study focuses on the stroma, the thickest layer of the cornea, accounting for 90% of its total thickness.^5^ The human stroma consists of superimposed lamellae ranging from 0.2 to 3 µm in thickness. These lamellae are composed of thin, well-organized collagen fibrils measuring 30 nm in diameter that are aligned in the same direction. They are roughly oriented along the inferior-superior and nasal-temporal axes with alternating orientations along the depth.^5^^,^^6^ This well-organized lamellar structure is disrupted in keratoconic corneas, as shown by several imaging techniques with complementary resolutions and fields of view: transmission electron microscopy,^7^^–^^9^ X-rays scattering,^10^^,^^11^ and optical coherence tomography (OCT).^12^^–^^15^

Keratoconic corneas have also been characterized using second harmonic generation (SHG) microscopy.^16^^–^^24^ This multiphoton modality enables fibrillar collagen imaging without exogenous staining, offering unmatched sensitivity and specificity. It has been demonstrated to yield unique three-dimensional (3D) images of the lamellar structure along the entire depth of intact control corneas.^6^^,^^25^^,^^26^ SHG studies of keratoconic corneas mostly focused on the anterior stroma and characterized the insertion of collagen lamellae to the Bowman's layer.^18^^–^^20^^,^^23^ They also reported an increased disorder in the lamellar structure.^16^^,^^17^^,^^20^^–^^24^ Nevertheless, these studies only looked at transverse sections or at the anterior stroma,^17^^–^^24^ or provided basic quantitative metrics of 3D images, thereby failing to fully capture the complex spatial organization of stromal alterations across the entire corneal thickness in keratoconus.^16^

In this study, we combine three complementary 3D imaging techniques and quantitative image analyses to provide a comprehensive characterization of stromal structural distortions in four human keratoconic corneas, across their full thickness, and in regions both proximal to and distant from the cone. Ultimately, such enhanced characterization may contribute to a better understanding of this pathology, triggering further studies that could enable earlier detection, more accurate staging, and improved patient management.

## Methods

### Human Cornea Samples

This study adhered to the tenets of the Declaration of Helsinki and followed international ethical requirements for human tissues. Handling of human corneas at the Laboratoire d'Optique et Biosciences, Palaiseau, France was declared to the French administration (CODECOH agreement DC-2018-3300).

Four keratoconic corneal buttons were obtained as surgical explants from the 15-20 ophthalmology hospital (Hôpital National de la Vision des Quinze-Vingt, Paris, France) (Table). All patients were included in the CCK-CONE cohort study after providing informed consent. Ethics committee approval was obtained from the Institut National des Données de Santé (no. 255645) after CEREES approval (13/02/2020). The data protection process agreed with the reference methodology MR-004 and the conformity declaration was approved by the CNIL (23/03/2020). The cohort protocol is available on the Health Data Hub.^27^ After surgery, the 8 mm diameter corneal buttons were preserved in a deturgescence and transport medium (CorneaJet; Eurobio Scientific, Les Ulis, France) for 30 minutes and then fixed in 4% paraformaldehyde (PFA) for 24 hours.

**Table.** Physiological and Clinical Data on the Human Corneas Under Study

Cornea	Patient Age	Gender	Post-surgery Interval to Fixation	Keratoconus Stage^*^	Minimal Thickness (µm, Without Epithelium)^†^
KC-1	51	F	30 minutes	Stage V	207
KC-2	63	F	30 minutes	Stage IV-V	150
KC-3	28	M	30 minutes	Stage IV	303
KC-4	43	M	30 minutes	Stage IV	234
			**Post-Mortem Interval**	**Reason for Rejection**	**Thickness**
**Cornea **	**Donor Age**	**Gender**	**to Collection**	**Transplantation**	**(µm, Without Epithelium)** ^#^

CC-1	59	M	<24 hours	Positive serology	604
CC-2	69	F	<24 hours	Poor EQ	490
CC-3	58	M	<24 hours	Poor EQ	542
CC-4	85	F	<24 hours	Poor EQ	474

CC, control cornea; EQ, endothelium quality; KC, keratoconic cornea.

* According to Sandali's classification.^14^

† In vivo measurement using SD-OCT

# Measurement on fixed cornea, using SHG.

Four human control corneas, which were unsuitable for transplantation and authorized for scientific use by the donor family, were obtained from the French Eye Bank (Banque Française des Yeux, Paris, France) (Table). They were preserved in an appropriate culture medium (CorneaMax; Eurobio Scientific) in an incubator set at 31°C, then placed for 48 hours in a cornea deturgescence medium without phenol red (StemAlpha 3 without phenol red; StemAlpha, Saint-Laurent-de-Chamousset, France), before fixation in 4% PFA for 24 hours at 4°C. To ensure that the corneal structure was not significantly impacted by the low endothelial quality or the different storage duration and process compared to keratoconic corneas, we verified that the thickness of all the control corneas was within the physiological range.

All fixed corneas were stored in 1% PFA at 4°C until imaging using a custom-built corneal chamber that enabled trans-detection of forward-directed signals.^6^^,^^28^ Briefly, the cornea was placed on a spherical lens adapted to its physiological curvature and clamped by a plastic ring. Ophthalmic gel (Lacrigel; Laboratoires Europhta, Monaco) was deposited on the corneas to ensure optical contact with the objective lens and to avoid cornea dehydration.^6^

### Spectral-Domain and Line-Field Confocal OCT Imaging

One day before surgery, patients underwent in vivo spectral-domain OCT (SD-OCT, RTVue‐100; Optovue Inc., Fremont, CA, USA). A series of 8 mm-wide scans with 5 µm axial and 15 µm transverse resolutions was acquired using the long corneal adapter module in the center of the cornea along different orientations to the physiological axes. Corneal thickness was computed to obtain 6-mm diameter pachymetry and epithelium maps.

Line-field confocal OCT (LC-OCT; DAMAE Medical, Paris, France) was used to image fixed corneas with high spatial resolution: 1.9 µm in the axial direction and 1.1 µm in the transverse one (Full Width at Half Maximum)^29^^–^^31^ (see Supplementary Material). Several two-dimensional transverse images and 3D volumes were recorded around the most damaged region of the keratoconic buttons, including the cone when this structure was visible. These regions were selected using a 2.5 mm diameter white-light image that is recorded in parallel to the OCT image in the LC-OCT device (Fig. 1). This helped identify the most relevant regions for further SHG imaging. Moreover, the ≈1 mm-wide LC-OCT images were compared to the series of 8-mm wide SD-OCT images acquired preoperatively in different orientations. Identifying similar patterns—specifically conic or fibrotic regions—made it possible to determine the orientation of the LC-OCT images with respect to the physiological axes.

**Figure 1.**
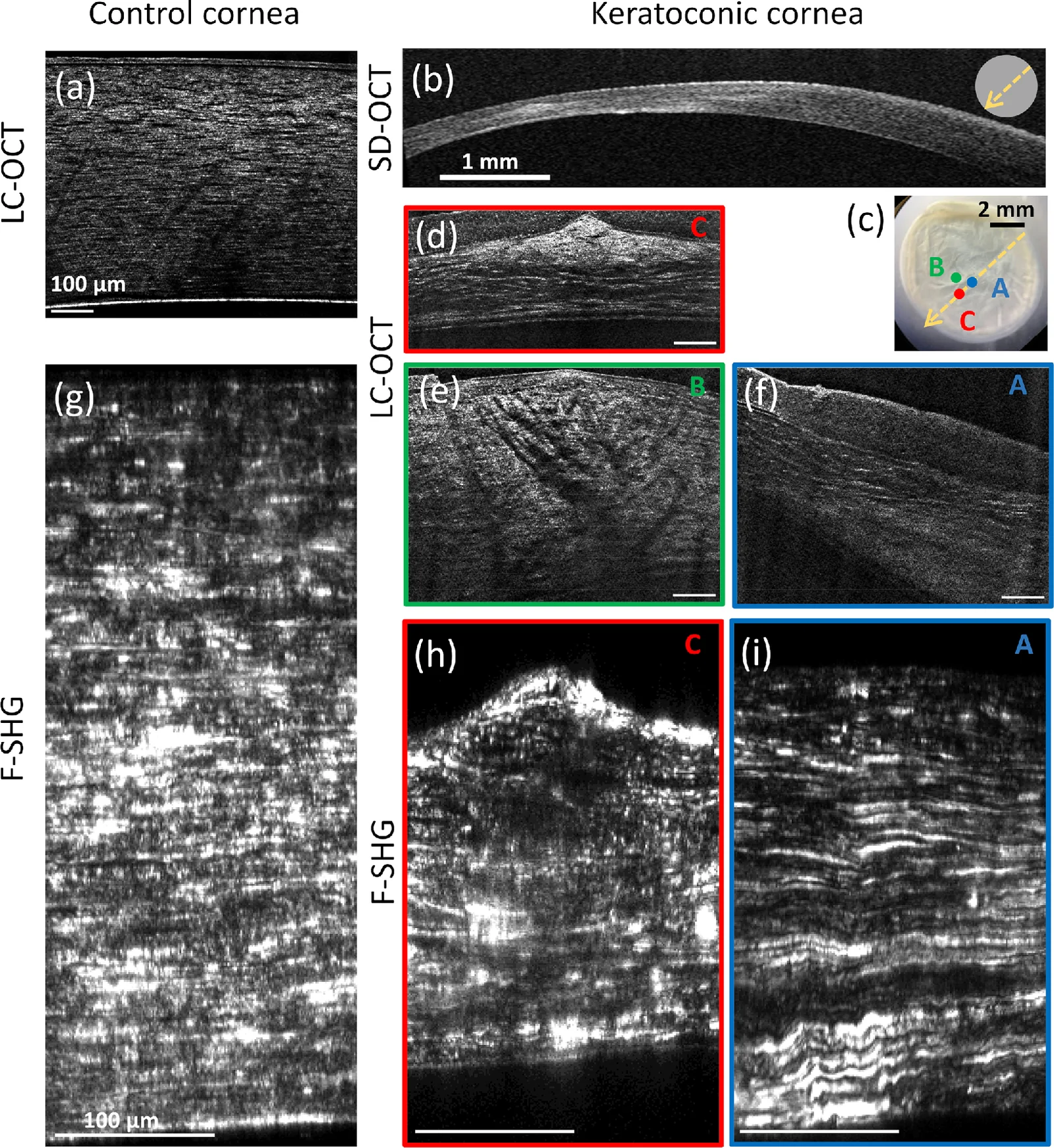
Clinical SD-OCT and LC-OCT images, and F-SHG transverse reslices of control (*CC-4*) and keratoconic (*KC-1*) corneas. **(a, d–f)** LC-OCT transverse images of control **(a)** and keratoconic **(d–f)** corneas. **(b)** In vivo SD-OCT image of keratoconic cornea before surgery. **(c)** White-light imaging of the keratoconic cornea: *C* indicates the cone region, as imaged in **(d)** and **(h)**, *A* and *B* indicate regions out of the cone, as imaged in **(e)**, **(f)**, and **(i)**. **(g–i)** Transverse reslices of en face forward-detected SHG images. The *yellow arrow* in **(b)** and **(c)** indicates the orientation of the OCT images to the physiological axes. *Scale bars*: 100 µm.

### SHG Microscopy

SHG imaging was performed on a custom-built upright laser-scanning multiphoton microscope as previously reported^6^^,^^28^ (See Supplementary Material). A 20×, 1.0 Numerical Aperture water-immersion objective lens (W Plan-Apochromat 20×/1.0 Korr DIC; Zeiss, Oberkochen, Germany) was used, achieving experimental transverse and axial resolutions (FWHM) of 0.35 µm × 1.36 µm². Z-stacks of forward-detected SHG images were recorded across the full thickness of the corneas at a pixel rate of 200 kHz, with a frame size of 195 × 195 pixels and voxel dimensions of 1.0 µm laterally and 1.0 or 2.0 µm axially. Several Z-stacks were recorded in each keratoconic corneas close to the position of the LC-OCT transverse images, at different distances from the cone (Fig. 1). The same experimental conditions were used to record several Z-stacks close to the apex of the four control corneas.

### Image Processing

The striated patterns observed in forward-detected SHG images^6^^,^^32^ were analyzed using the ImageJ plugins OrientationJ^33^ and TWOMBLI.^34^ These plugins were applied to the Z-stacks after each image was normalized to its maximum, and they used the same parameters across all images.

OrientationJ provided the orientation in each pixel and a parameter in the [0,1] range, called the coherency, used to assess the quality of the orientation determination.^33^ The main orientation in each en face image was also computed at each depth in the SHG stack, as well as polar histograms of all voxel orientations in a Z-stack.

TWOMBLI^34^ provided a segmentation of the striations, as well as the following metrics (Supplementary Material):

– *Align* measures the extent to which striations on the SHG image are oriented in a similar direction. It ranges between 0 (not aligned) and 1 (perfectly aligned in the same direction).
– *NormEnd* measures the number of end points along the striations on the SHG image. It is normalized to the total length of the segmented striations in the image. This metric is larger for interrupted striations (dotted lines) and smaller for continuous striations.
– *End/Branch* measures the number of end points normalized to the number of striations crossings that is smaller for intertwined striations and larger for parallel striations.

### Statistical Analysis

Statistical tests were performed using GraphPad Prism (GraphPad, San Diego, CA, USA). The metrics computed on all images of the different Z-stacks recorded in the same cornea were first averaged since these images are not independent. Unpaired *t*-tests with Welch corrections were performed on the average values and the *P*-values were corrected for multiple comparisons using the Holm-Sidak method. Adjusted *P*-values are displayed as non-significant (ns) when *P* > 0.05, * when *P* < 0.05, ** when *P* < 0.01, and *** when *P* < 0.005.

## Results

### Clinical Imaging of Keratoconic Corneas

SD-OCT images of the anterior segment were recorded before surgery (Fig. 1.b). The position of the cone was assessed using the topographic maps (Supplementary Figs. S1–S4). The SD-OCT images show a markedly thinned and ectatic cornea. Significant stromal scarring and fibrosis are observed, which lead to increased reflectivity and irregularity of the corneal layers. The epithelial layer appears irregular: it thickens over areas where the stroma is most thinned, while the peripheral regions maintain a more normal thickness. The posterior corneal surface appears distorted, while the central corneal thickness is severely reduced. The corneal architecture is disrupted consistent with chronic scarring after keratoconus progression.

### LC-OCT and SHG Imaging of Control and Keratoconic Corneas

Control corneas from Eye Bank and keratoconic buttons after surgery were imaged using LC-OCT to obtain transverse images and 3D volumes of their morphology at the micrometer scale (Fig. 1). Control corneas show as expected hyperreflective lines parallel to the corneal surface, which correspond to the anterior and posterior interfaces, the epithelium-Bowman's layer interface and the keratocytes within the stroma (Fig. 1a). Stromal striae appear as hyporeflective oblique lines starting from the endothelium.^15^ LC-OCT images of keratoconic corneas are recorded in several regions of interest, both inside and outside the conic zone (Figs. 1d–f), as shown in the white-light image (Fig. 1c). The cone is clearly seen as a hyperreflective conic region, indicative of fibrosis (Fig. 1d). The epithelium is thickened around the cone, whereas the stroma is thinner compared to control corneas (Figs. 1d, 1e). The anterior stroma shows regions with thick hyporeflective and hyperreflective undulated lines (Figs. 1d, 1e). Outside the cone, stromal striae are more numerous than in control corneas and they are oriented in more vertical directions (Fig. 1f). These observations are consistent in all keratoconic corneas under study (Supplementary Figs. S1–S4).

Then, en face forward-detected SHG images were recorded. Transverse reslices are displayed for comparison with LC-OCT images and show the collagen lamellae parallel to the corneal surface in the stroma (Figs. 1g–i). They appear less straight in the keratoconic corneas than in the control ones. In the cone, they appear shorter. These SHG transverse reslices also confirm that the stroma is thinner in the keratoconic corneas than in the control ones. *En face* SHG images show domains of striated patterns with the same orientation that corresponds to lamellae composed of collagen fibrils orientated along the striations (Figs. 2a–c, 2g–i).^6^^,^^25^^,^^32^ The striations in the control corneas are straight and continuous lines, and the size of the domains increases in the posterior stroma. In contrast, the striations in the keratoconic corneas are interrupted and wavy, and the size of the domains is larger than in the control corneas, regardless of depth.

**Figure 2.**
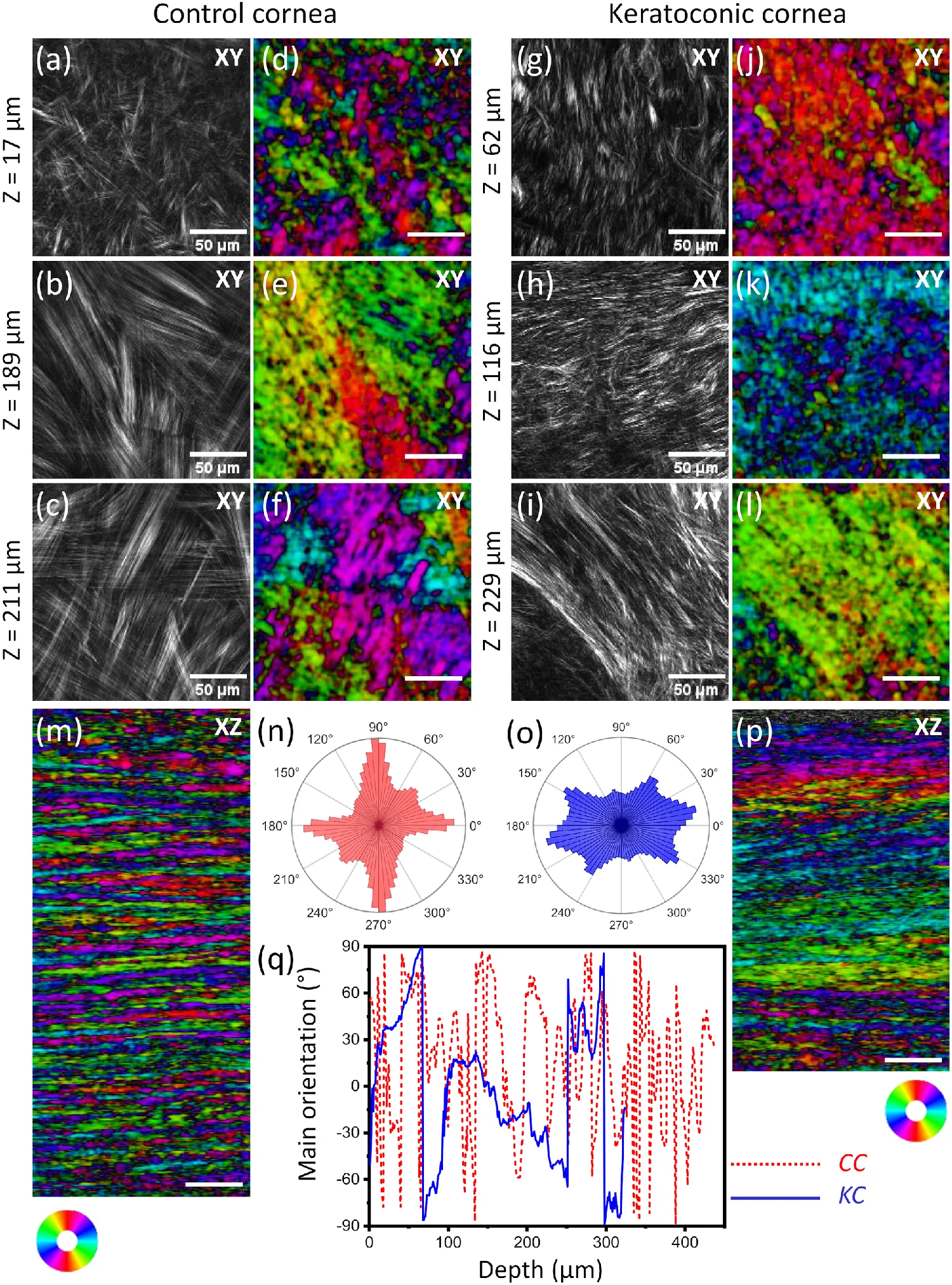
Orientation of lamellar domains in control (*CC-4*) and keratoconic (*KC-4*) corneas (one specific Z-stack per cornea). **(a–c, g–i)** En face forward-detected SHG images and **(d–f, j–l)** the corresponding orientation maps obtained using OrientationJ of **(a–f)** a control cornea and **(g–l)** a keratoconic one. **(m, p)** Transverse reslices of the orientation map of **(m)** the control cornea and **(p)** the keratoconic one. **(n, o)** Polar plots of the orientation histograms across the full depth in **(n)** the control cornea and **(o)** the keratoconic one. **(q)** Depth profile of the main orientation in the control and keratoconic corneas. Note that the abrupt change from +90° and −86° in the depth profile of the keratoconic corneas corresponds to a slight change in orientation (4°), because SHG imaging is not sensitive to polarity. *Scale bar*: 50 µm. Orientation maps are displayed using HSV look-up table: *H* codes the orientation as displayed in the color wheel (*bottom right*), *S* = 1, and *V* is the coherency. The orientation is in the [−90° to 90°] range, with 90° being equivalent to −90°. XZ transverse reslices show the orientation in the XY plane.

### Orientation Analyses

Extraction of the orientation of the striated patterns in en face SHG images (Figs. 2d–f, 2j–l) confirms that, in the control corneas, domains with similar orientations are small in the anterior stroma and increase in size with depth. In contrast, they are larger at every depth in the keratoconic corneas. Transverse reslices also show strong differences between the control and keratoconic corneas (Figs. 2m, 2p). The control corneas show superimposed thin lamellar domains with different orientations, whereas the keratoconic cornea exhibits similar orientations in thicker regions. This is clearly observed in the depth profiles of the main orientations in each Z-plane (Fig. 2q). The main orientation changes progressively in the keratoconic corneas, whereas the control corneas show alternating thin domains with different orientations. Finally, histograms of the orientations in all voxels along the full depth of the corneas show that the collagen is oriented along two main orthogonal orientations in the control corneas, whereas it is distributed in a single wide angular band in the keratoconic corneas (Figs. 2n, 2o). These observations are consistent across the four keratoconic corneas under study (Supplementary Figs. S5–S7). However, the orientation of the single angular band varies among them, and no clear behavior emerges.

### Quantitation of SHG Striated Patterns as a Function of the Distance to the Cone

Analyses of the en face forward-detected SHG images show straighter and more continuous segmented striations in the control corneas than in the keratoconic corneas across the full thickness of the stroma (Fig. 3). To examine any potential structural differences as a function of the distance to the cone, the striated patterns are analyzed in several Z-stacks recorded along a line crossing the cone of a keratoconic cornea (Fig. 4a): within the cone, close to the cone (distance ≈ 0.5 mm), and 2.5 mm away from it. The distributions of three metrics of interest are compared to those of a control cornea (Figs. 4b–d). First, the metric *Align* is similar to that of a control cornea in the region farthest from the cone. It increases closer to the cone and within the cone, but with widely spread values. Second, the metric *NormEnd* is larger than that of the control cornea in all regions of the keratoconic cornea and it shows similar values in all these regions regardless of their distance to the cone. The same result is obtained with the third metric, *End/Branch*.

**Figure 3.**
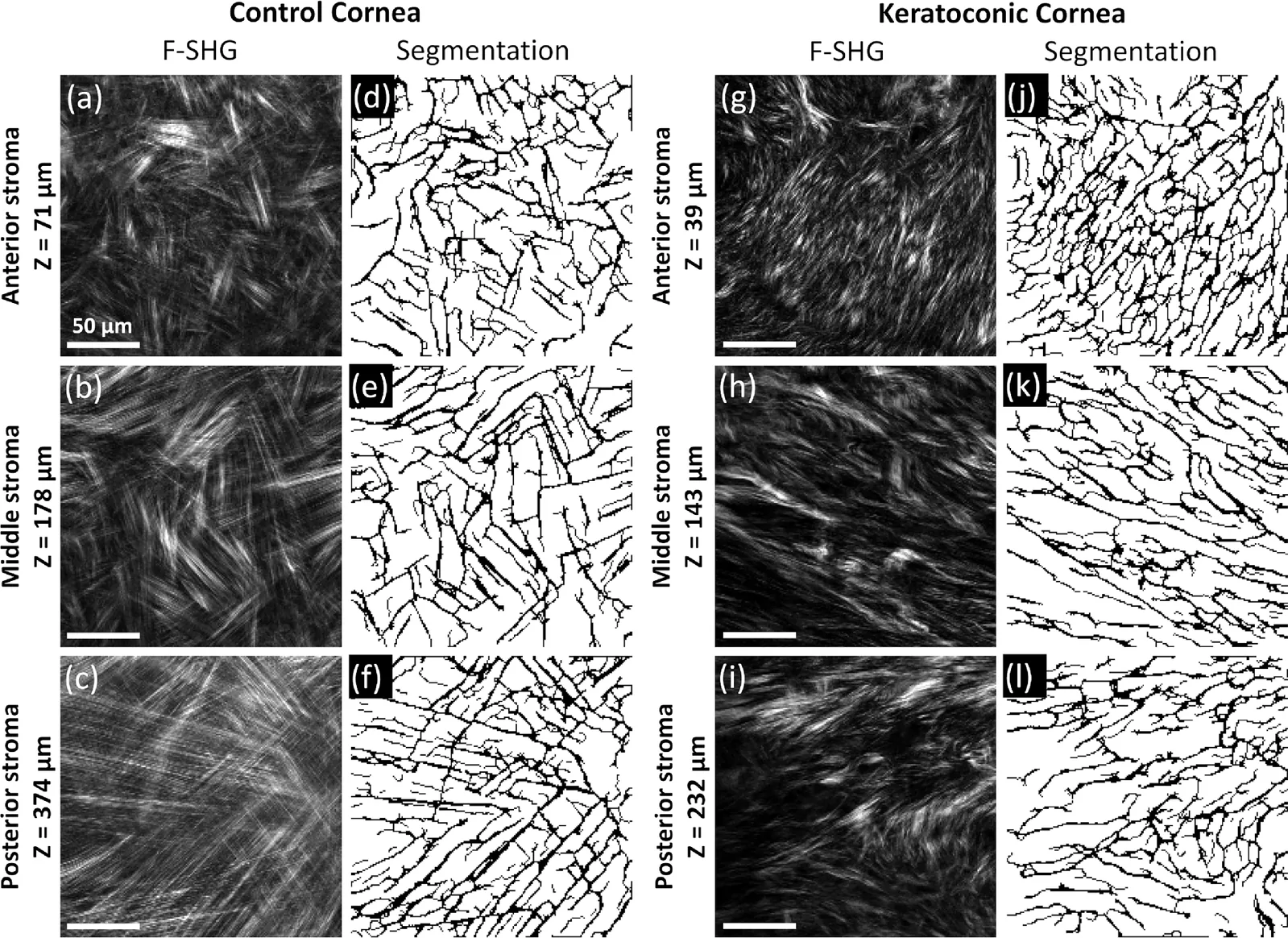
Segmentation of striated patterns in control (*CC-1*) and keratoconic (*KC-3*) corneas. **(a–c, g–i)** En face forward-detected SHG images and **(d–f, g–i)** the corresponding segmented images obtained using Twombli, in a control cornea **(a–f)** and a keratoconic one **(g–l)**. Images are shown in the anterior **(a, d, g, j)**, resp. middle **(b, e, h, k)**, resp. posterior **(c, f, i, l)** stroma. *Scale bar*: 50 µm. SHG images are normalized to their maximum and the contrast is slightly enhanced (range, 10–200).

**Figure 4.**
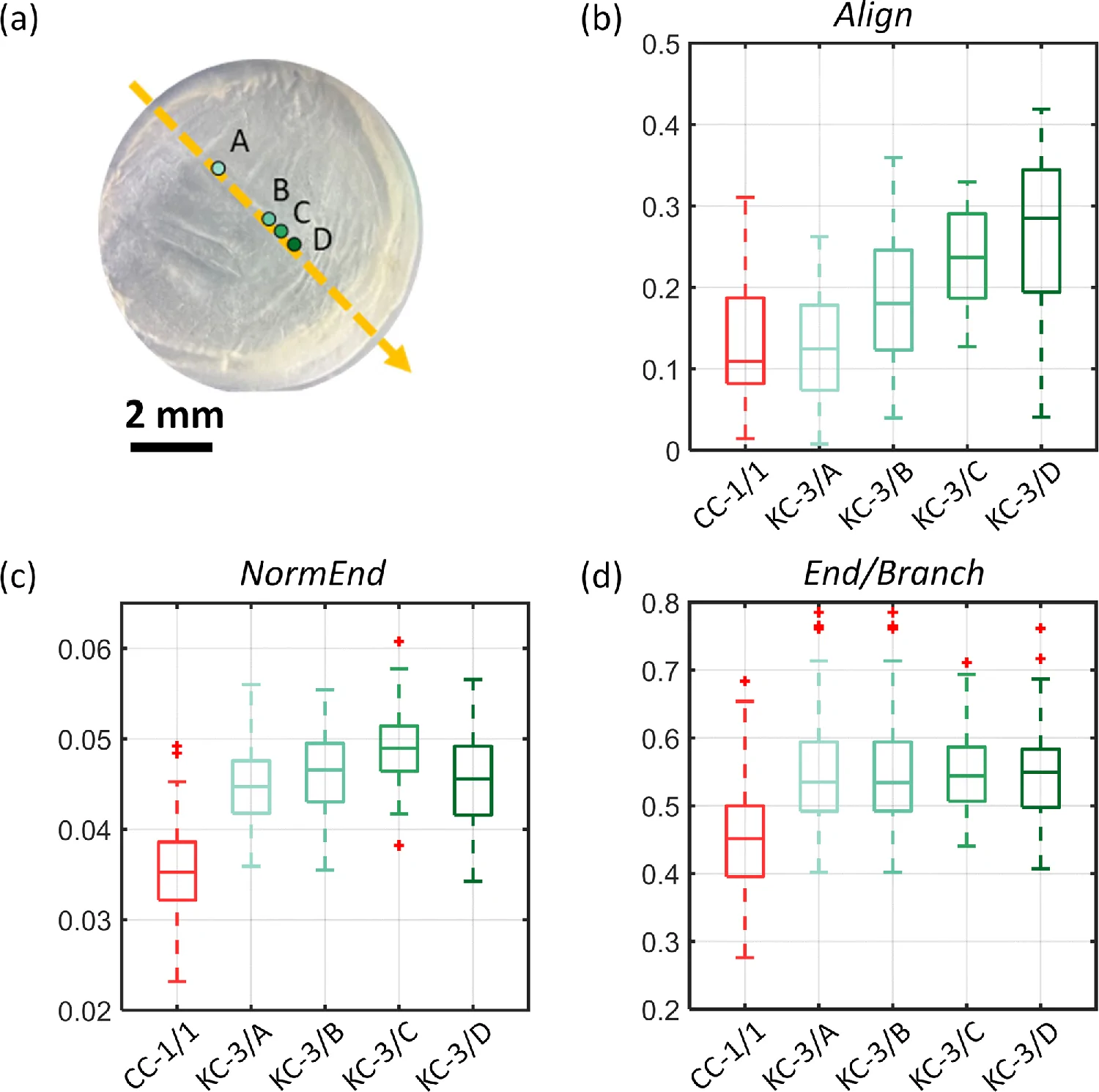
Quantitation of SHG striated patterns in a keratoconic cornea (*KC-3*) as a function of the distance to the cone. **(a)** View of the keratoconic cornea. Region *C* is the conical region, regions *B* and *D* are located 0.5 mm away from the cone, and region *A* is located 2.5 mm from the cone. **(b–d)** Median boxplots of **(b)** *Align* metric, **(c)** *NormEnd* metric, and **(c)** *End/Branch* metric for a control cornea (CC-1) and the four regions of interest of the keratoconic cornea (KC-3). Every box corresponds to one Z-stack with typically 200 images for KC-3 and 450 images for CC-1.

A similar analysis is performed on the anterior stroma, the middle stroma and the posterior stroma to examine possible structural differences as a function of the depth (Supplementary Fig. S8). For this purpose, each Z-stack of en face SHG images is divided in three equal parts based on depth. These data show the same results as in the entire stroma.

### Quantitative Comparison of SHG Striated Patterns in Control and Keratoconic Corneas

Next, we examine any potential structural differences between control corneas and keratoconic corneas. Figures 5a–c display the distribution of the three metrics across the depth of three Z-stacks for each cornea, except only two Z-stack for KC-1. First, the *Align* metric is larger for keratoconic corneas than for control ones (Fig. 5a). It means that striated patterns in each en face forward-detected SHG image are better aligned together in keratoconic corneas than in control corneas, which is consistent with our qualitative observation. Nevertheless, this difference is not statistically significant (Adjusted *P* = 0.075, Fig. 5d). Second, the *NormEnd* metric is larger for keratoconic corneas than for control ones, with clearly separated distributions, as expected from our qualitative observations of interrupted striations in the keratoconic corneas and continuous striations in the control corneas (Fig. 5b). This difference is significant (Adjusted *P* = 0.008, Fig. 5d). Similarly, the third metric, *End/Branch*, is larger for keratoconic corneas than for control ones, as expected from our qualitative observations of parallel striations in the keratoconic corneas and intertwined striations in the control corneas (Fig. 5c). This difference is significant (Adjusted *P* = 0.015, Fig. 5d).

**Figure 5.**
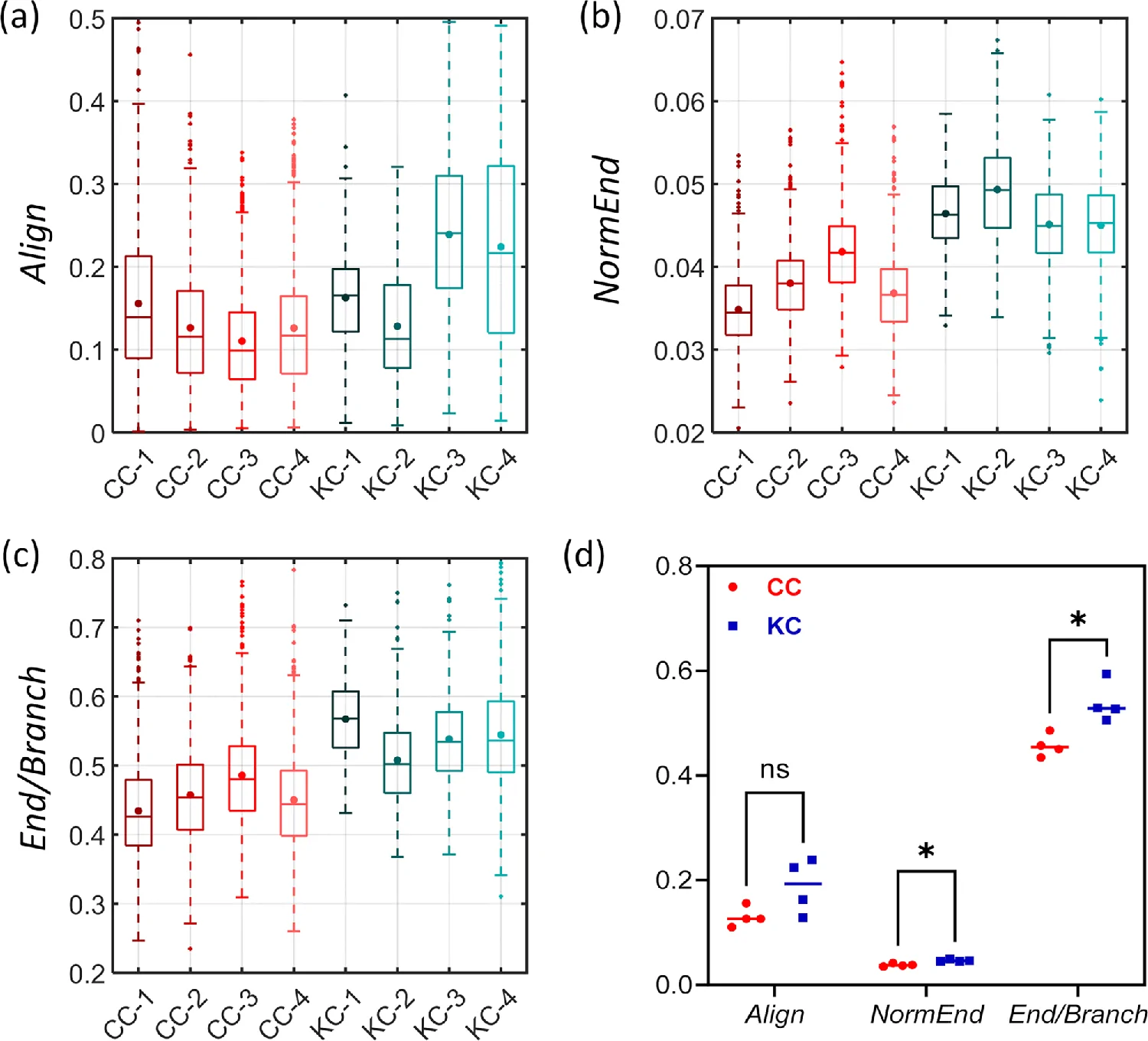
Quantitation of SHG striated patterns in control (*CC*) and keratoconic (*KC*) corneas. **(a–c)** Median boxplots of **(a)** *Align* metric, **(b)** *NormEnd* metric, and **(c)** *End/Branch* metric along three Z-stacks for every cornea (except only 2 Z-stacks for KC-1). The distributions correspond to approximately 980 and 450 images from the three Z-stacks of the control and keratoconic corneas (CC-1: 1215, CC-2: 866, CC-3: 816, CC-4: 1038, KC-1: 97, KC-2: 337, KC-3:654, KC-4:728). The mean is plotted as a *solid circle*. **(d)** Statistical tests based on mean values in each type of cornea.

Similar analyses performed on the anterior, middle and posterior stroma shows results similar to those of the entire stroma. Nevertheless, the *Align* statistical test is significant in the anterior stroma (Supplementary Fig. S9).

## Discussion

In this study, we extract a set of quantitative structural metrics that reveal distinct and spatially resolved differences between control and keratoconic corneas. Although control corneas from the Eye Bank display low endothelial quality and are not processed in the same way as keratoconic corneas, these limitations are mitigated by verifying that they all exhibit a thickness within the physiological range, thereby allowing their use as controls in our study. Our multimodal and multiscale approach using complementary optical techniques enables a detailed characterization of stromal remodeling, improving our understanding of the structural mechanisms underlying keratoconus progression. In vivo imaging before surgery using a clinical SD-OCT device enables classification of the pathological stage, as well as subsequent localization and orientation of post-surgery images using LC-OCT and SHG devices, which offer higher spatial resolution at the expense of a reduced field of view. Notably, stromal striae appear more clearly in LC-OCT images than in SD-OCT images, which allows us to confirm previous reports about changes to these striae in keratoconic corneas.^15^ A well-controlled imaging protocol and a dedicated custom-build chamber are then used to record LC-OCT and SHG images in the same regions of each cornea. Advantageously, SHG signal is obtained only from fibrillar collagen, revealing all the collagen lamellae within the stroma^6^^,^^32^ (Supplementary Material), whereas OCT signal arises from fibrotic collagen and keratocytes around the collagen lamellae, not from the collagen lamellae themselves.^15^ Because of this specificity to collagen, our series of 3D SHG images confirms previous reports on the lamellar structure of control corneas^6^^,^^25^^,^^32^ and provides unique structural data on the organization of stromal lamellae in keratoconic buttons over their full depth and at controlled distances from the conical region.

Many image analysis tools have been developed to quantify SHG images.^24^^,^^35^ In this study, two well-documented ImageJ plugins are used to analyze the striated patterns observed in the en face SHG images.^33^^,^^34^ First, TWOMBLI segments the striated patterns and extracts quantitative parameters that characterize these patterns in each image. In contrast to the straight lines observed in control corneas, interrupted lines with numerous branching points are observed in the keratoconic corneas. These dotted striations in the keratoconic corneas are attributed to wavy lamellae, going in and out of the imaging plane. It is consistent with the transverse reconstructions that directly show these wavy lamellae, as well as LC-OCT transverse images showing similar features. A key advantage of SHG images here is the ability to process quantitative metrics and explore the extent of these distortions. As expected, significant differences are observed between control and keratoconic corneas in the normalized number of end points along the striations and in the normalized number of endpoints relative to the number of striations crossings. In contrast, similar values are obtained regardless of the distance to the cone in a keratoconic cornea, indicating that the lamellae distortion extends beyond the conical region. More SHG images in more regions and more corneas, including less advanced keratoconus, would be necessary to confirm this result. Nevertheless, it aligns with previous reports indicating thinning and ultrastructural alterations in the central and peripheral stroma of keratoconic corneas.^9^^,^^36^^,^^37^ It supports the hypothesis of a global corneal alteration potentially related to genetic or other systemic factors, rather than a purely local alteration due to mechanical stress in case of eye rubbing or other local processes.^2^ This suggests that keratoconus should be considered a multifactorial disease involving intrinsic abnormalities of the corneal extracellular matrix, including altered collagen synthesis, organization, and turnover.^38^^,^^39^ Several studies have reported dysregulation of matrix metalloproteinases, increased oxidative stress, and reduced expression of collagen cross-linking enzymes, all of which may contribute to stromal weakening.^40^ In addition, genetic predisposition has been supported by familial aggregation and the identification of susceptibility loci associated with corneal structure and biomechanics.^41^ Systemic associations, such as connective tissue disorders (e.g., Ehlers-Danlos syndrome or Marfan syndrome), further reinforce the concept of a generalized extracellular matrix dysfunction.^42^^,^^43^ Although environmental factors such as chronic eye rubbing and atopy may exacerbate disease progression, they are likely to act as secondary triggers superimposed on an underlying structural fragility of the cornea.^44^^,^^45^

Second, orientation maps and the main orientations at each depth are computed using OrientationJ. The orientation maps show that the lamellae are wider in keratoconic than in control corneas. Additionally, transverse reconstructions of the main orientation in each two-dimensional en face image, along with their depth profiles, show that the lamellae are much thicker. Most importantly, the plywood structure of healthy corneas is completely lost. Instead of alternating thin lamellae with nearly orthogonal orientations as observed in control corneas, the keratoconic corneas exhibit slight progressive changes in collagen orientation, demonstrating a complete reorganization of stromal collagen. The resulting orientation distributions across the full depth of the stroma are irregular and show a single wide angular band, in contrast with the two orthogonal peaks observed in control corneas. This result aligns with previous studies that reported similar distortion of the global orientations distributions obtained from x-ray scattering^10^^,^^11^ and Fourier transform analyses of en face SHG images.^21^ However, none of these studies provide en face orientation maps or depth profiles of the main orientation, limiting their ability to fully characterize the lamellar organization. Efficient visualization of the lamellar series can be obtained through electron microscopy, a technique that previous studies have used to map full transverse sections of healthy and keratoconic corneas.^7^^,^^46^ These studies have shown that the lamellar structure is still present in keratoconic corneas, although the anterior interwoven lamellae are lost and the lamellae are thicker in the posterior stroma and thinner in the anterior stroma compared to healthy corneas.^7^^,^^46^ In light of these ultrastructural characterizations, we thus attribute our results to a series of superimposed lamellae with slight shifts in orientation between them. This reveals that, although the lamellar structure is preserved, its spatial organization is profoundly altered. Importantly, SHG microscopy combined with advanced orientation analysis uniquely enables this 3D depth-resolved characterization of collagen organization across the full corneal thickness. This capability provides unprecedented insight into stromal remodeling in keratoconus and opens new perspectives for quantitative assessment of disease severity and progression.

This distorted lamellar structure may explain the deterioration of the mechanical properties of keratoconic corneas as measured in routine clinical practice by use of Corvis ST (Oculus, Wetzlar, Germany) or Ocular Response Analyzer (ORA, Reichert Ophthalmic Instruments Inc., Buffalo, NY, USA). Indeed, the stiffness of healthy corneas is attributed to their plywood structure composed of alternating thin lamellae with orthogonal orientations, creating an almost isotropic material.^47^^,^^48^ Accordingly, the alignment of lamellae along the two orthogonal directions is reinforced in an inflation assay when the cornea is submitted to a pressure larger than the physiological intra-ocular pressure.^49^ In contrast, the uniaxial structure composed of wide, thick, and undulated lamellae observed in keratoconus is consistent with lower stiffness. First, lamellae appear undulated in the keratoconic cornea, meaning they need to unfold prior providing significant stress, and are thus softer. Second, their anisotropic distribution of orientation implies that the stroma will be easier to deform in the perpendicular direction, which has been shown to favor bulging shape.^50^ Moreover, these structural changes can be associated to a decrease in cohesion between collagen fibrils and the noncollagenous matrix,^11^ or to a decrease in the lamellae branching points^20^ resulting in subsequent fibrillar and lamellar slippage and orientational redistribution, rather than to the loss of lamellae.^51^ This slippage mechanism also explains possible plastic, irreversible deformation of the cornea under pressure. Note also that these structural changes challenge the efficiency of cross-linking treatments pending the therapeutic effect arises mainly from cross-links formation rather than myofibroblast formation with stiff collagen secretion.^52^^–^^54^ Although they can limit the progression of the pathology, they cannot fully restore the mechanical properties because they only fix the uniaxial orientation distribution and do not realign the lamellae in two perpendicular directions.^55^

Our findings were obtained from a series of only four keratoconic corneas in an advanced pathological stage (stages IV to V in the Sandali's classification). We expect the structural distortions observed in this pilot exploratory series to be confirmed in additional regions and samples, and generalized to earlier stages of the pathology, albeit to a lesser extent. However, this would be difficult to investigate using in vivo SHG microscopy because, although in vivo implementation is possible, it requires compromises that result in less resolved images that do not clearly show the striated features.^56^ We therefore propose probing these structural changes by measuring the related increase in the corneal birefringence. Such a measure of the corneal birefringence has already been implemented using OCT or scanning laser polarimetry and could easily be incorporated into clinical devices.^57^^,^^58^ This new approach to probing the early stages of keratoconus would complement biomechanical measures using Corvis or ORA and should hopefully improve the early diagnosis of this pathology.

## Supplementary Material

Supplement 1

## References

[1] Krachmer JH, Feder RS, Belin MW. Keratoconus and related noninflammatory corneal thinning disorders. *Surv Ophthalmol*. 1984; 28: 293–322. 10.1016/0039-6257(84)90094-8 6230745

[2] Santodomingo-Rubido J, Carracedo G, Suzaki A, Villa-Collar C, Vincent SJ, Wolffsohn JS. Keratoconus: an updated review. *Cont Lens Anterior Eye*. 2022; 45(3): 101559. 10.1016/j.clae.2021.101559 34991971

[3] Hashemi H, Heydarian S, Hooshmand E, et al. The prevalence and risk factors for keratoconus: a systematic review and meta-analysis. *Cornea*. 2020; 39: 263–270. 10.1097/ICO.0000000000002150 31498247

[4] Dunker SL, Armitage WJ, Armitage M, et al. Practice patterns of corneal transplantation in Europe: first report by the European Cornea and Cell Transplantation Registry. *J Cataract Refract Surg*. 2021; 47: 865–869. 10.1097/j.jcrs.0000000000000574 33577274

[5] Krachmer J, Mannis M, Holland E. *Cornea* *-* 3rd edition. Philadelphia: Mosby Elsevier; 2011.

[6] Raoux C, Chessel A, Mahou P, Latour G, Schanne-Klein M-C. Unveiling the lamellar structure of the human cornea over its full thickness using polarization-resolved SHG microscopy. *Light Sci Appl*. 2023; 12: 190. 10.1038/s41377-023-01224-0 PMC10394036 37528091

[7] Mathew JH, Goosey JD, Soderberg PG, Bergmanson JP. Lamellar changes in the keratoconic cornea. *Acta Ophthalmol*. 2015; 93: 767–773. 10.1111/aos.12811 26278201

[8] Akhtar S, Bron AJ, Salvi SM, Hawksworth NR, Tuft SJ, Meek KM. Ultrastructural analysis of collagen fibrils and proteoglycans in keratoconus. *Acta Ophthalmol*. 2008; 86: 764–772. 10.1111/j.1755-3768.2007.01142.x 18422999

[9] Alkanaan A, Barsotti R, Kirat O, Almubrad T, Khan A, Akhtar S. Ultrastructural study of peripheral and central stroma of keratoconus cornea. *Br J Ophthalmol*. 2017; 101: 845–850. 10.1136/bjophthalmol-2016-309834 28416495

[10] Daxer A, Fratzl P. Collagen fibril orientation in the human corneal stroma and its implication in keratoconus. *Invest Ophthalmol Vis Sci*. 1997; 38: 121–129. 9008637

[11] Meek KM, Tuft SJ, Huang Y, et al. Changes in collagen orientation and distribution in keratoconus corneas. *Invest Ophthalmol Vis Sci*. 2005; 46: 1948–1956. 10.1167/iovs.04-1253 15914608

[12] Hollingsworth JG, Efron N. Observations of banding patterns (Vogt striae) in keratoconus: a confocal microscopy study. *Cornea*. 2005; 24: 162–166. 10.1097/01.ico.0000141231.03225.d8 15725884

[13] Mocan MC, Yilmaz PT, Irkec M, Orhan M. The significance of Vogt's striae in keratoconus as evaluated by in vivo confocal microscopy. *Clin Exp Ophthalmol*. 2008; 36: 329–334. 10.1111/j.1442-9071.2008.01737.x 18700919

[14] Sandali O, El Sanharawi M, Temstet C, et al. Fourier-domain optical coherence tomography imaging in keratoconus: a corneal structural classification. *Ophthalmology*. 2013; 120: 2403–2412. 10.1016/j.ophtha.2013.05.027 23932599

[15] Grieve K, Ghoubay D, Georgeon C, et al. Stromal striae: a new insight into corneal physiology and mechanics. *Sci Rep*. 2017; 7: 13584. 10.1038/s41598-017-13194-6 PMC5648881 29051516

[16] Lo W, Chen WL, Hsueh CM, et al. Fast Fourier transform-based analysis of second-harmonic generation image in keratoconic cornea. *Invest Ophthalmol Vis Sci*. 2012; 53: 3501–3507. 10.1167/iovs.10-6697 22570347

[17] Tan HY, Sun Y, Lo W, et al. Multiphoton fluorescence and second harmonic generation imaging of the structural alterations in keratoconus ex vivo. *Invest Ophthalmol Vis Sci*. 2006; 47: 5251–5259. 10.1167/iovs.06-0386 17122110

[18] Morishige N, Shin-gyou-uchi R, Azumi H, et al. Quantitative analysis of collagen lamellae in the normal and keratoconic human cornea by second harmonic generation imaging microscopy. *Invest Ophth Vis Sci*. 2014; 55: 8377–8385. 10.1167/iovs.14-15348 25425311

[19] Mercatelli R, Ratto F, Rossi F, et al. Three-dimensional mapping of the orientation of collagen corneal lamellae in healthy and keratoconic human corneas using SHG microscopy. *J Biophot*. 2017; 10: 75–83. 10.1002/jbio.201600122 27472438

[20] Mikula E, Winkler M, Juhasz T, et al. Axial mechanical and structural characterization of keratoconus corneas. *Exp Eye Res*. 2018; 175: 14–19. 10.1016/j.exer.2018.05.019 PMC7324026 29842851

[21] Batista A, Breunig HG, Konig A, et al. High-resolution, label-free two-photon imaging of diseased human corneas. *J Biomed Opt*. 2018; 23(3): 036002. 10.1117/1.JBO.23.3.036002 29500874

[22] Avila FJ, Artal P, Bueno JM. Quantitative discrimination of healthy and diseased corneas with second harmonic generation microscopy. *Transl Vis Sci Technol*. 2019; 8(3): 51. 10.1167/tvst.8.3.51 PMC6601709 31293806

[23] Raoux C, Schmeltz M, Bied M, et al. Quantitative structural imaging of keratoconic corneas using polarization-resolved SHG microscopy. *Biomed Opt Express*. 2021; 12: 4163–4178. 10.1364/BOE.426145 PMC8367248 34457406

[24] Guimaraes P, Morgado M, Batista A. On the quantitative analysis of lamellar collagen arrangement with second-harmonic generation imaging. *Biomed Opt Express*. 2024; 15: 2666–2680. 10.1364/BOE.516817 PMC11019681 38633085

[25] Aptel F, Olivier N, Deniset-Besseau A, et al. Multimodal nonlinear imaging of the human cornea. *Invest Ophthalmol Vis Sci*. 2010; 51: 2459–2465. 10.1167/iovs.09-4586 20071677

[26] Batista A, Guimaraes P, Domingues JP, Quadrado MJ, Morgado AM. Two-photon imaging for non-invasive corneal examination. *Sensors*. 2022; 22(24): 9699. 10.3390/s22249699 PMC9783858 36560071

[27] Health Data Hub. Available at: https://www.health-data-hub.fr/projets/observatoire-de-la-prise-en-charge-des-patients-presentant-un-keratocone-du-centre-de.

[28] Nyembo Kasongo P, Mahou P, Sintès J-M, Latour G, Schanne-Klein M-C. In situ determination and matching of the refractive index of the human cornea to improve polarization-resolved SHG imaging in depth. *Biomed Opt Express*. 2025; 16: 3270–3282. 10.1364/BOE.564209 PMC12339295 40809962

[29] Dubois A, Levecq O, Azimani H, et al. Line-field confocal time-domain optical coherence tomography with dynamic focusing. *Opt Express*. 2018; 26: 33534–33542. 10.1364/OE.26.033534 30650800

[30] Ogien J, Levecq O, Azimani H, Dubois A. Dual-mode line-field confocal optical coherence tomography for ultrahigh-resolution vertical and horizontal section imaging of human skin in vivo. *Biomed Opt Express*. 2020; 11: 1327–1335. 10.1364/BOE.385303 PMC7075601 32206413

[31] Galante G, Vilbert M, Desvois L, et al. Non-invasive quantitative investigation of varnish stratigraphy in historical artifacts using line-field confocal OCT. *Heritage Sci*. 2026; 14: 193.

[32] Latour G, Gusachenko I, Kowalczuk L, Lamarre I, Schanne-Klein M-C. In vivo structural imaging of the cornea by polarization-resolved Second Harmonic microscopy. *Biomed Opt Express*. 2012; 3(1): 1. 10.1364/BOE.3.000001 PMC3255328 22254163

[33] Puspoki Z, Storath M, Sage D, Unser M. Transforms and operators for directional bioimage analysis: a survey. *Adv Anat Embryol Cell Biol*. 2016; 219: 69–93. 10.1007/978-3-319-28549-8_3 27207363

[34] Wershof E, Park D, Barry DJ, et al. A FIJI macro for quantifying pattern in extracellular matrix. *Life Science Alliance*. 2021; 4(3): e202000880. 10.26508/lsa.202000880 PMC7898596 33504622

[35] Nejim Z, Navarro L, Morin C, Badel P. Quantitative analysis of second harmonic generated images of collagen fibers: a review. *Res Biomed Eng*. 2023; 39: 273–295.

[36] Brautaset RL, Nilsson M, Miller WL, Leach NE, Tukler JH, Bergmanson JP. Central and peripheral corneal thinning in keratoconus. *Cornea*. 2013; 32: 257–261. 10.1097/ICO.0b013e31825240d7 22562062

[37] Schlatter B, Beck M, Frueh BE, Tappeiner C, Zinkernagel M. Evaluation of scleral and corneal thickness in keratoconus patients. *J Cataract Refract Surg*. 2015; 41: 1073–1080. 10.1016/j.jcrs.2014.08.035 25935338

[38] Rong SS, Ma STU, Yu XT, et al. Genetic associations for keratoconus: a systematic review and meta-analysis. *Sci Rep*. 2017; 7(1): 4620. 10.1038/s41598-017-04393-2 PMC5496893 28676647

[39] Monteiro de Barros MR, Chakravarti S. Pathogenesis of keratoconus: NRF2-antioxidant, extracellular matrix and cellular dysfunctions. *Exp Eye Res*. 2022; 219: 109062. 10.1016/j.exer.2022.109062 PMC12056795 35385756

[40] Navel V, Malecaze J, Pereira B, et al. Oxidative and antioxidative stress markers in keratoconus: a systematic review and meta-analysis. *Acta Ophthalmol*. 2021; 99(6): e777–e794. 10.1111/aos.14714 33354927

[41] Hardcastle AJ, Liskova P, Bykhovskaya Y, et al. A multi-ethnic genome-wide association study implicates collagen matrix integrity and cell differentiation pathways in keratoconus. *Commun Biol*. 2021; 4: 266. 10.1038/s42003-021-01784-0 PMC7921564 33649486

[42] Robati RM, Einollahi B, Einollahi H, Younespour S, Fadaifard S. Skin biophysical characteristics in patients with keratoconus: a controlled study. *Scientifica*. 2016; 2016: 6789081. 10.1155/2016/6789081 PMC4925989 27403376

[43] Kling S, Torres-Netto EA, Abdshahzadeh H, Espana EM, Hafezi F. Collagen V insufficiency in a mouse model for Ehlers Danlos-syndrome affects viscoelastic biomechanical properties explaining thin and brittle corneas. *Sci Rep*. 2021; 11(1): 17362. 10.1038/s41598-021-96775-w PMC8405695 34462473

[44] Gordon-Shaag A, Millodot M, Shneor E, Liu Y. The genetic and environmental factors for keratoconus. *Biomed Res Int*. 2015; 2015: 795738. 10.1155/2015/795738 PMC4449900 26075261

[45] Yang K, Li D, Xu L, Pang C, Zhao D, Ren S. Independent and interactive effects of eye rubbing and atopy on keratoconus. *Front in Immunol*. 2022; 13: 999435. 10.3389/fimmu.2022.999435 PMC9558125 36248837

[46] Mathew JH, Bergmanson JP, Doughty MJ. Fine structure of the interface between the anterior limiting lamina and the anterior stromal fibrils of the human cornea. *Invest Ophthalmol Vis Sci*. 2008; 49: 3914–3918. 10.1167/iovs.07-0707 18765633

[47] Boote C, Dennis S, Huang Y, Quantock AJ, Meek KM. Lamellar orientation in human cornea in relation to mechanical properties. *J Struct Biol*. 2005; 149: 1–6. 10.1016/j.jsb.2004.08.009 15629652

[48] Elsheikh A, Alhasso D. Mechanical anisotropy of porcine cornea and correlation with stromal microstructure. *Exp Eye Res*. 2009; 88: 1084–1091. 10.1016/j.exer.2009.01.010 19450454

[49] Benoit A, Latour G, Schanne-Klein M-C, Allain J-M. Simultaneous microstructural and mechanical characterization of human corneas at increasing pressure. *J Mech Behav Biomed Mater*. 2016; 60: 93–105. 10.1016/j.jmbbm.2015.12.031 26773650

[50] Gefen A, Shalom R, Elad D, Mandel Y. Biomechanical analysis of the keratoconic cornea. *J Mech Behav Biomed Mater*. 2009; 2: 224–236. 10.1016/j.jmbbm.2008.07.002 19627827

[51] Padmanabhan P, Elsheikh A. Keratoconus: a biomechanical perspective. *Curr Eye Res*. 2023; 48: 121–129. 10.1080/02713683.2022.2088798 35746888

[52] Bradford SM, Mikula ER, Juhasz T, Brown DJ, Jester JV. Collagen fiber crimping following in vivo UVA-induced corneal crosslinking. *Exp Eye Res*. 2018; 177: 173–180. 10.1016/j.exer.2018.08.009 PMC6911705 30118656

[53] Bueno JM, Avila FJ, Martinez-Garcia MC. Quantitative Analysis of the Corneal Collagen Distribution after In Vivo Cross-Linking with Second Harmonic Microscopy. *Biomed Res Int*. 2019; 2019: 3860498. 10.1155/2019/3860498 PMC6348900 30756083

[54] Zeitoune AA, Bersanetti PA, Schor P, Erbes LA, Cesar CL, Adur J. Comparison of morphological changes of corneal collagen fibers treated with collagen crosslinking agents using second harmonic generation images. *Int J Biol Macromol*. 2020; 165(Pt A): 346–353. 10.1016/j.ijbiomac.2020.09.147 32987082

[55] Borderie VM, Georgeon C, Louissi N, et al. Corvis(ST) biomechanical indices in the diagnosis of corneal stromal and endothelial disorders: an artificial intelligence-based comparative study. *Br J Ophthalmol*. 2026; 110: 396–402. 10.1136/bjo-2025-327855 PMC13018791 41130662

[56] Avila FJ, Gambin A, Artal P, Bueno JM. In vivo two-photon microscopy of the human eye. *Sci Rep*. 2019; 9(1): 10121. 10.1038/s41598-019-46568-z PMC6626016 31300680

[57] Irsch K, Shah AA. Birefringence of the central cornea in children assessed with scanning laser polarimetry. *J Biomed Opt*. 2012; 17(8): 086001. 10.1117/1.JBO.17.8.086001 23224188

[58] Li Q, Karnowski K, Untracht G, et al. Vectorial birefringence imaging by optical coherence microscopy for assessing fibrillar microstructures in the cornea and limbus. *Biomed Opt Express*. 2020; 11: 1122–1138. 10.1364/BOE.382755 PMC7041478 32206403

