# Supplementary material for: Quantitation of Stromal Distortion Across the Full Depth of Keratoconic Corneas
Source: Invest Ophthalmol Vis Sci. 2026 Oct 23;67(12):58. doi: 10.1167/iovs.67.12.58 (PMC13715923; doi:10.1167/iovs.67.12.58)
Supplement: Supplementary file 1 [file iovs-67-12-58_s001.pdf]

Quantitation of stromal distortion across the full depth of keratoconic corneas

Supplementary Material

Poncia Nyembo Kasongo,[1] Djida Ghoubay,[2] Jean-Marc Allain,[3] Malika Hamrani,[2] Cristina Georgeon,[2] Vincent Borderie,[2] Gaël Latour,[1,4,†] and Marie-Claire Schanne-Klein[1,†]

[1] Laboratoire d'Optique et Biosciences, CNRS, Inserm, École Polytechnique, Institut Polytechnique de Paris, Palaiseau, France
[2] Sorbonne Université, Hôpital National de la Vision des 15-20, INSERM, Institut de la Vision, GRC32, CIC15-20, F-75012 Paris, France
[3] Laboratoire de mécanique des solides, CNRS, École polytechnique, Institut Polytechnique de Paris, Palaiseau, France
[4] Université Paris-Saclay, 91190 Gif-sur-Yvette, France
[†] *These authors contributed equally.*

Supplementary Methods

Linefield confocal optical coherence tomography (LC-OCT)

LC-OCT is an optical microscopy technique that combines the advantages of confocal microscopy, in which light is spatially selected by conjugating the focal volume and the detection, and optical coherence tomography, in which light is selected based on the temporal coherence properties. Therefore, it provides a better signal-to-noise ratio in depth within biological tissues [1,2]. This technique was initially developed by DAMAE Medical (Paris, France) for dermatological applications and was designed for use in contact mode with the skin [2,3]. In collaboration with DAMAE Medical, we have developed a setup specifically adapted for non-contact water immersion imaging of biological tissues.[4] This device enables water immersion imaging with a working distance of approximately 1 mm. The experimental spatial resolution is 1.9 µm in the axial direction and 1.1 µm in the lateral one (Full Width at Half Maximum). The field of view is 1200 (x, length) × 500 (y, width) × 700 (z, depth) μm^3. The motorized micrometer stage enables

measurements of the distance between series of images recorded in the same cornea. The acquisition frame rate is 3 Hz allowing real-time 2D image recording. The acquisition time of a full volume is around 2.5 minutes.

Second Harmonic Generation (SHG) microscopy

SHG imaging was performed on a custom-built upright laser-scanning multiphoton microscope equipped with a Titanium-Sapphire laser set at 860 nm (Mai-Tai, Spectra-Physics) as previously reported.[5,6] A 20x, 1.0 Numerical Aperture (NA) water-immersion objective lens (W Plan-Apochromat 20×/1.0 Korr DIC, Zeiss) was used. The lateral and axial resolutions (Full Width at Half Maximum) were measured as 0.35 µm × 1.36 µm² using KTP nanocrystals deposited on a glass slide[7] and the full Field of View (FOV) is 634 × 634 µm². Multiphoton signals were detected in both the backward and forward directions using photon-counting photomultiplier tubes (P25PC, Electron Tubes, UK). Dichroic mirrors (FF-458-Di01, Semrock) were used to separate SHG from 2PEF signals and appropriate spectral filters were used to reject laser excitation (FF01-680/SP and FF01-720/SP, Semrock) and select SHG signal (FF01-427/10 interferential filter, Semrock).

As demonstrated in previous reports [5,8], the striated patterns observed in *en face* forward-detected SHG images reflect the direction of the collagen fibrils within lamellae. Regions that emit SHG correspond to parallel fibrils within lamellae, while regions with no SHG signal correspond to antiparallel fibrils, which form a centrosymmetric material in which SHG is forbidden. Note that these striated patterns are only observed in forward-detected SHG images, whereas backward-detected images are homogeneous because of the smaller coherence length in reflection. Polarization-resolved SHG imaging is then necessary to measure collagen orientation in a backward geometry [8,9]. This technique has been demonstrated to be efficient and accurate, though

being slow. Unfortunately, it is sensitive to the birefringence of uniformly oriented collagen assemblies and may produce inaccurate results in cases of high corneal structural distortion.[10] Thus, SHG imaging, combined with quantitative image analyses, appears to be the most reliable and fastest method for characterizing keratoconic buttons. Furthermore, the specificity of SHG images to collagen facilitates quantitative analyses because no segmentation is needed.

Image processing

The striated patterns were analyzed using the ImageJ plugins: OrientationJ [11] and TWOMBLI. [12] OrientationJ calculated the gradient structure tensor in local windows and provided the local orientation as the eigenvector of the larger eigenvalue of this tensor. [11] OrientationJ also calculated the normalized difference of the 2 eigenvalues to estimate whether the larger eigenvalue corresponded to a dominant orientation. This parameter, which is in the [0,1] range, is called the coherency and was used to assess the quality of the orientation determination. The main orientation in each *en face* image was also computed at each depth in the SHG stack as the orientation obtained from the average of all local gradient structure tensors within the image. In our study, we used the following parameters for all images: local window size of 1 pixel and cubic spline gradient. We then plotted the orientation maps using an Hue-Saturation-Value (HSV) color map where H coded the orientation, S=1 and V was the coherency. We also plotted polar histograms of all voxel orientations in a Z-stack. The polar histograms of the keratoconic corneas were oriented to the physiological axes. The polar histograms of the control corneas were plotted with their 2 main orientation directions along the physiological axes as expected from previous reports [5].

TWOMBLI [12] relies on existing ImageJ plugins such as Ridge Detection, Anamorph and OrientationJ. It was used with the following parameters in our study: contrast saturation = 0.5, min

line width = 2, max line width = 10, Min branch length = 2, max display HDM = 255. This automated pipeline provided a segmentation of the striations as well as a broad range of quantitative metrics that were relevant to quantify the pattern of collagen SHG images:

- Total Length of segmented striations, called *TotLength* in the following. It is given in pixels and must be compared to the total number of pixels in every SHG image: 195x195=38 025. This metric was used to select the SHG segmented images with enough segmented striations. SHG images with *TotLength < 2800 (7.4%)* were considered as non-significant and filtered out.
- Alignment, called *Align* in the following: this metric is calculated from the global gradient structure tensor and measures the extent to which striations on the SHG image are oriented in a similar direction. It ranges between 0 (not aligned) and 1 (perfectly aligned in the same direction).
- Normalized number of end points, called *NormEnd* in the following: this metric measures the number of end points along the striations on the SHG image. It is normalized to the total length of the segmented striations in the image (*TotLength)*. This metric is larger for interrupted striations (dotted lines) and smaller for continuous striations.

References

1. Dubois A, Levecq O, Azimani H, et al. Line-field confocal time-domain optical coherence tomography with dynamic focusing. *Opt Express*. Dec 24 2018;26(26):33534-33542. doi:10.1364/OE.26.033534

2. Ogien J, Levecq O, Azimani H, Dubois A. Dual-mode line-field confocal optical coherence tomography for ultrahigh-resolution vertical and horizontal section imaging of human skin in vivo. *Biomed Opt Express*. Mar 1 2020;11(3):1327-1335. doi:10.1364/BOE.385303
3. Latriglia F, Ogien J, Tavernier C, et al. Line-Field Confocal Optical Coherence Tomography (LC-OCT) for Skin Imaging in Dermatology. *Life (Basel)*. Nov 28 2023;13(12)doi:10.3390/life13122268
4. Galante G, Vilbert M, Desvois L, et al. Non-invasive quantitative investigation of varnish stratigraphy in historical artifacts using line-field confocal OCT. *Heritage Science*. 2026;14:193. doi:https://doi.org/10.1038/s40494-026-02460-4
5. Raoux C, Chessel A, Mahou P, Latour G, Schanne-Klein M-C. Unveiling the lamellar structure of the human cornea over its full thickness using polarization-resolved SHG microscopy. *Light: Science and Applications*. 2023;12(1):190. doi:https://doi.org/10.1038/s41377-023-01224-0
6. Nyembo Kasongo P, Mahou P, Sintès J-M, Latour G, Schanne-Klein M-C. In situ determination and matching of the refractive index of the human cornea to improve polarization-resolved SHG imaging in depth. *Biomedical Optics Express*. 2025/08/01 2025;16(8):3270-3282. doi:10.1364/BOE.564209
7. Mahou P, Malkinson G, Chaudan E, Gacoin T, Beaurepaire E, Supatto W. Metrology of Multiphoton Microscopes Using Second Harmonic Generation Nanoprobes. *Small*. Nov 13 2017;13(42)doi:10.1002/smll.201701442
8. Latour G, Gusachenko I, Kowalczuk L, Lamarre I, Schanne-Klein M-C. *In vivo* structural imaging of the cornea by polarization-resolved Second Harmonic microscopy. *Biomed Opt Express*. 2012;3(1):1. doi:10.1364/BOE.3.000001

9. Raoux C, Schmeltz M, Bied M, et al. Quantitative structural imaging of keratoconic corneas using polarization-resolved SHG microscopy. *Biomed Opt Express*. 2021;12(7):4163-4178. doi:https://doi.org/10.1364/BOE.426145

10. Gusachenko I, Latour G, Schanne-Klein M-C. Polarization-resolved Second Harmonic microscopy in anisotropic thick tissues. *Opt Express*. 2010;18(18):19339-19352.

11. Puspoki Z, Storath M, Sage D, Unser M. Transforms and Operators for Directional Bioimage Analysis: A Survey. *Focus on Bio-Image Informatics, Adv Anat Embryol Cell Biol*. 2016:69-93.

12. Wershof E, Park D, Barry DJ, et al. A FIJI macro for quantifying pattern in extracellular matrix. *Life Science Alliance*. 2021;4(3):e202000880. doi:10.26508/lsa.202000880

Supplementary Figures

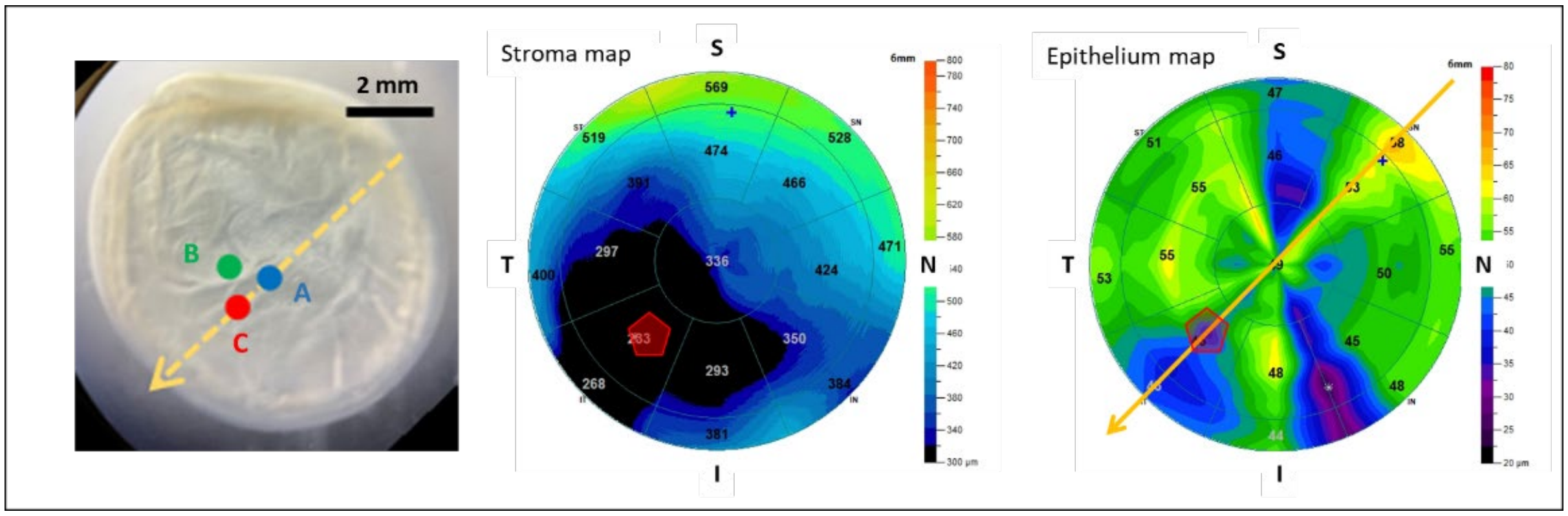

Supplementary Figure S1. Topography of keratoconic cornea KC1. (left) White light image of the button showing the positions of the LC-OCT and SHG images. (center, right) 6mm-diameter topography maps of the stroma and the epithelium. The red pentagon shows the estimated position of the cone. The orange line shows the orientation of the SD-OCT image and the x axis of the LC-OCT and SHG images. N: nasal, T: temporal, S: superior, I: inferior.

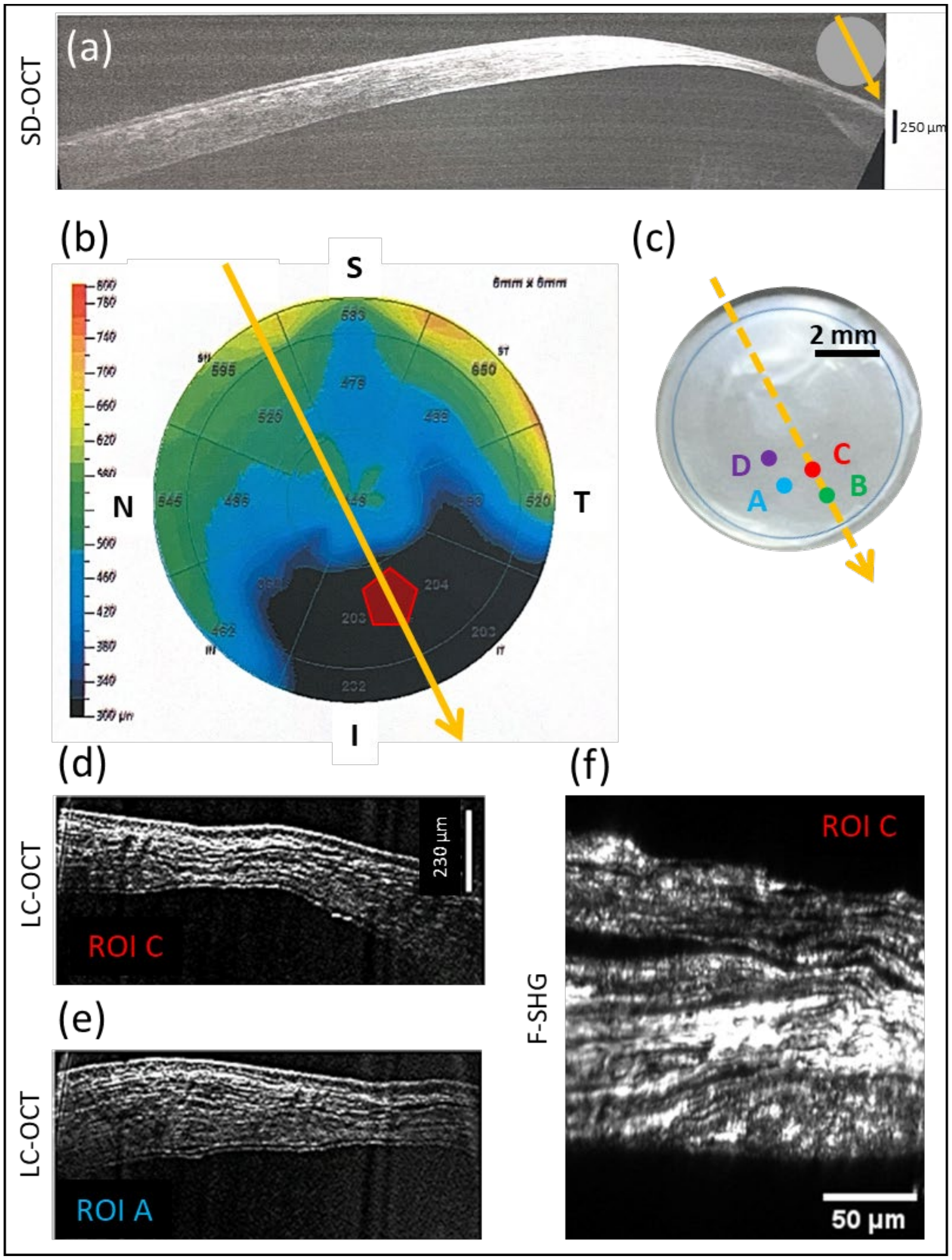

Supplementary Figure S2. Topography and images of keratoconic cornea KC2. (a) SD-OCT image; (b) 6-mm wide pachymetry map. The red pentagon shows the estimated position of the cone. The orange line shows the orientation of the SD-OCT image and the x axis of the LC-OCT and SHG images. N: nasal, T: temporal, S: superior, I: inferior. (c) White-light image of the corneal button showing the position of the ROIs. (d,e) LC-OCT images of ROIs C and A. (f) SHG transverse reslices of the ROI C.

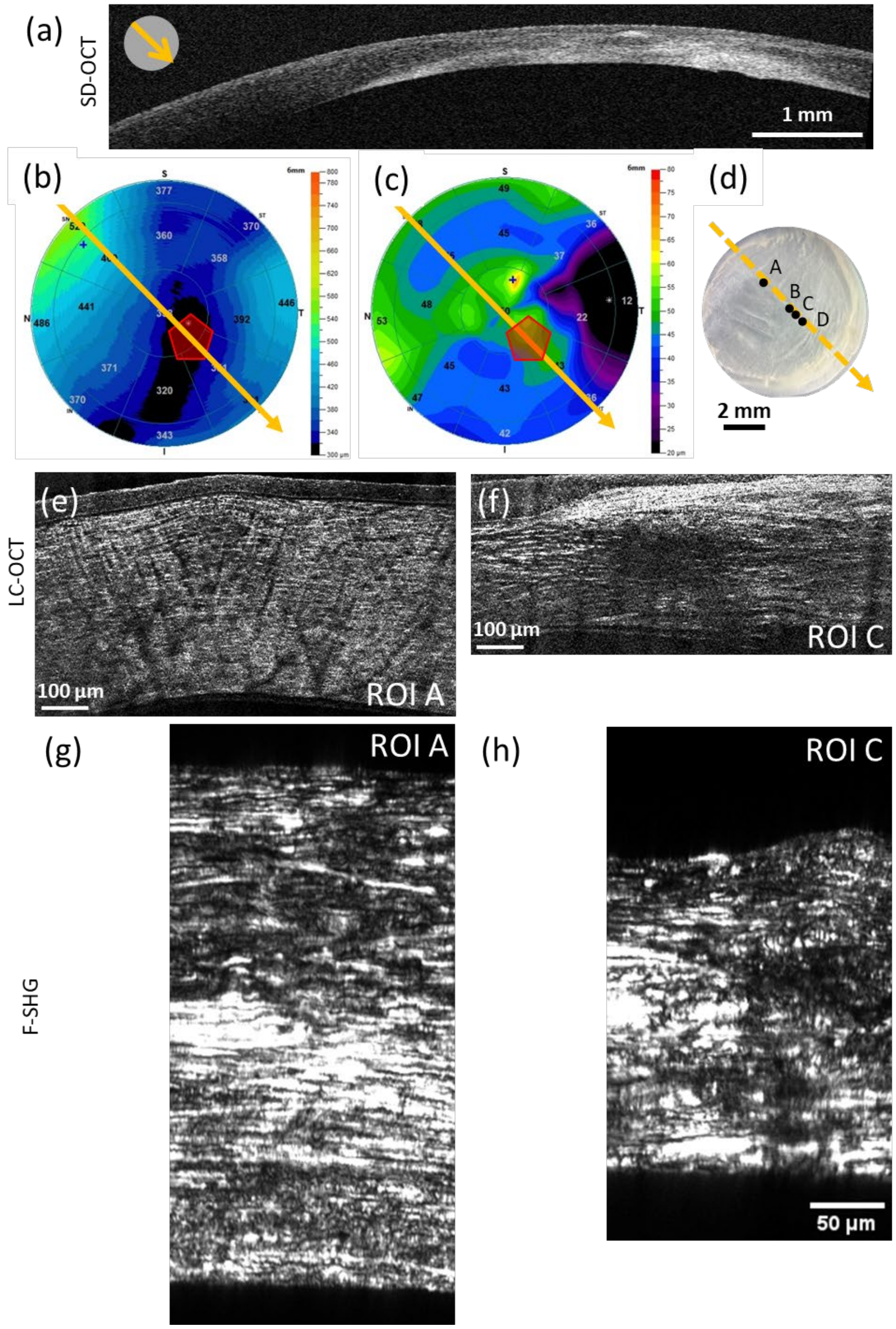

Supplementary Figure S3. Topography and images of keratoconic cornea KC3. (a) SD-OCT image; (b,c) 6-mm wide pachymetry map. The red pentagon shows the estimated position of the cone. The orange line shows the orientation of the SD-OCT image and the x axis of the LC-OCT and SHG images. N: nasal, T: temporal, S: superior, I: inferior. (d) White-light image of the corneal button showing the position of the ROIs. (e,f) LC-OCT images of ROIs A and C. (g,h) SHG transverse reslices of the ROIs A and C.

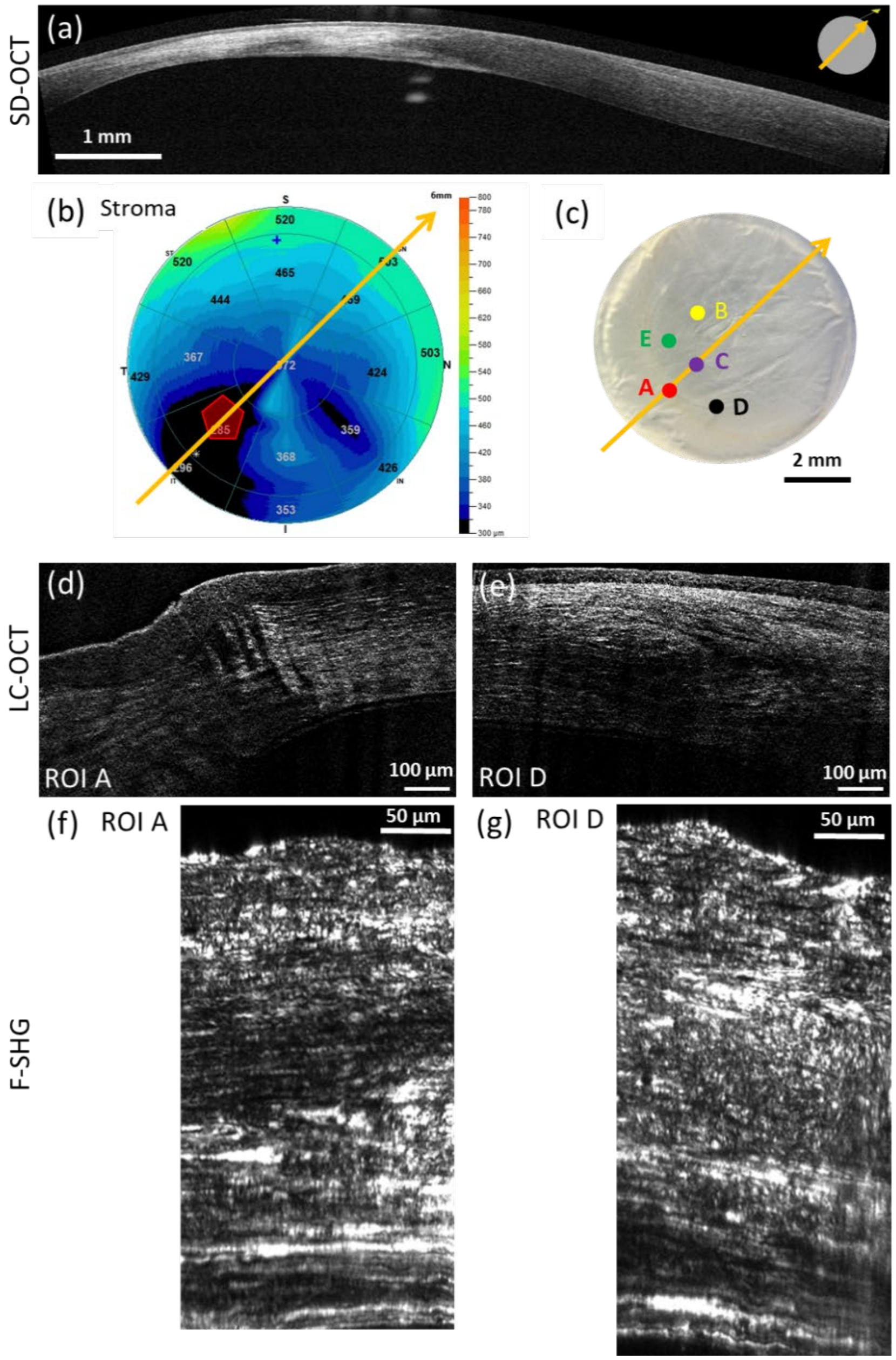

Supplementary Figure S4. Topography and images of keratoconic cornea KC4. (a) SD-OCT image; (b) 6-mm wide pachymetry map. The red pentagon shows the estimated position of the cone. The orange line shows the orientation of the SD-OCT image and the x axis of the LC-OCT and SHG images. N: nasal, T: temporal, S: superior, I: inferior. (c) White-light image of the corneal button showing the position of the ROIs. (d,e) LC-OCT images of ROIs A and D. (f, g) SHG transverse reslices of the ROIs A and D.

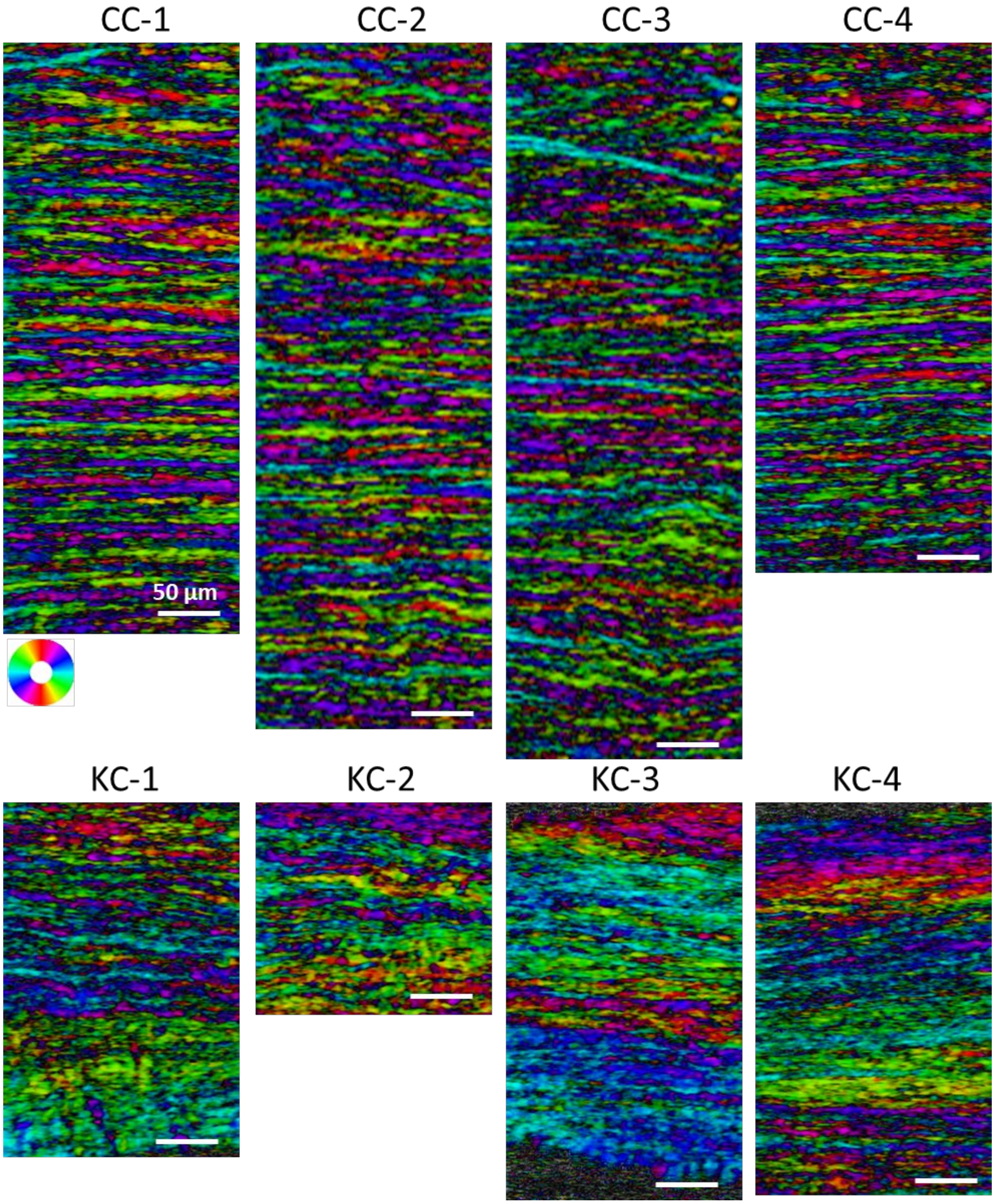

Supplementary Figure S5. Transverse reslices of orientation maps obtained from one Z-stack for each cornea using OrientationJ. Scale bar:50 µm

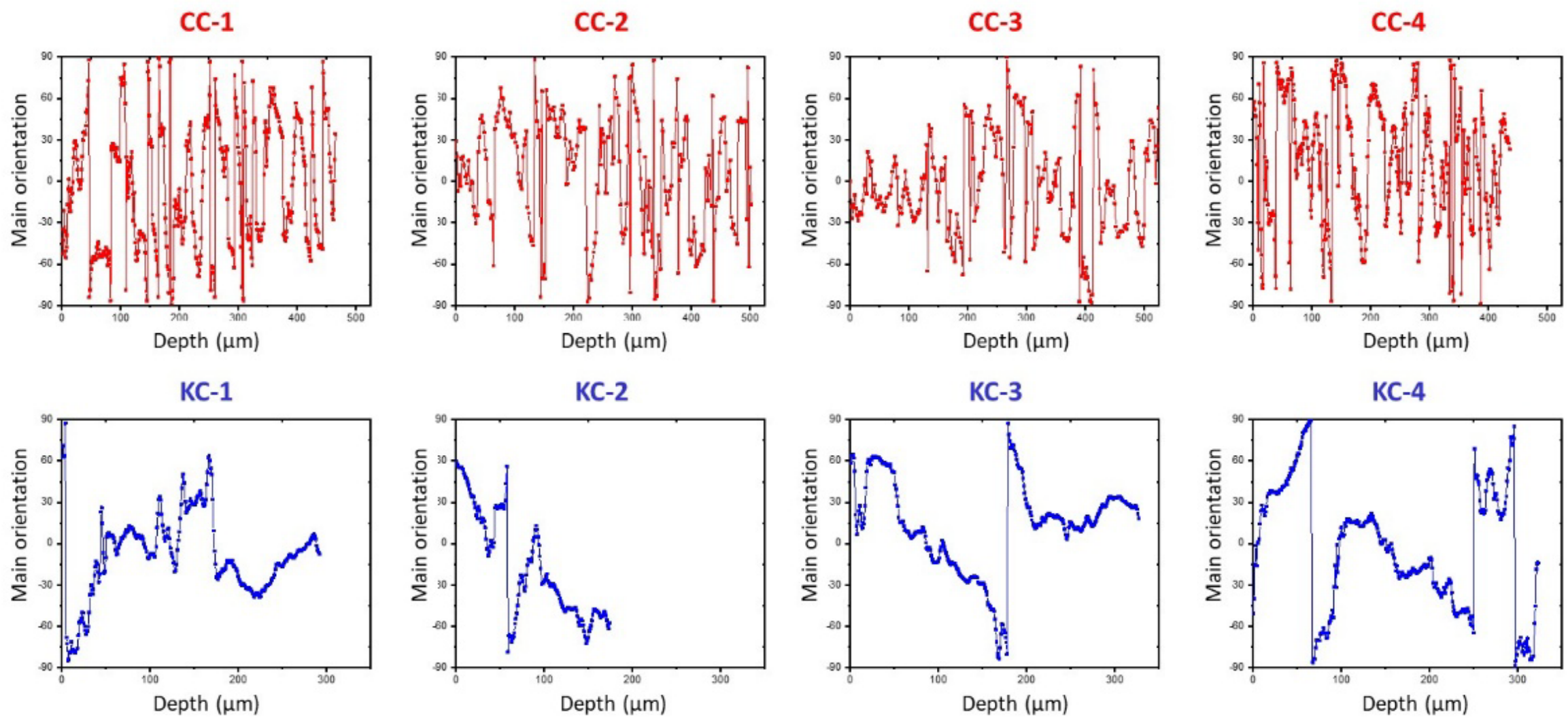

Supplementary Figure S6. Depth profiles of main orientation obtained from one Z-stack for each cornea using OrientationJ

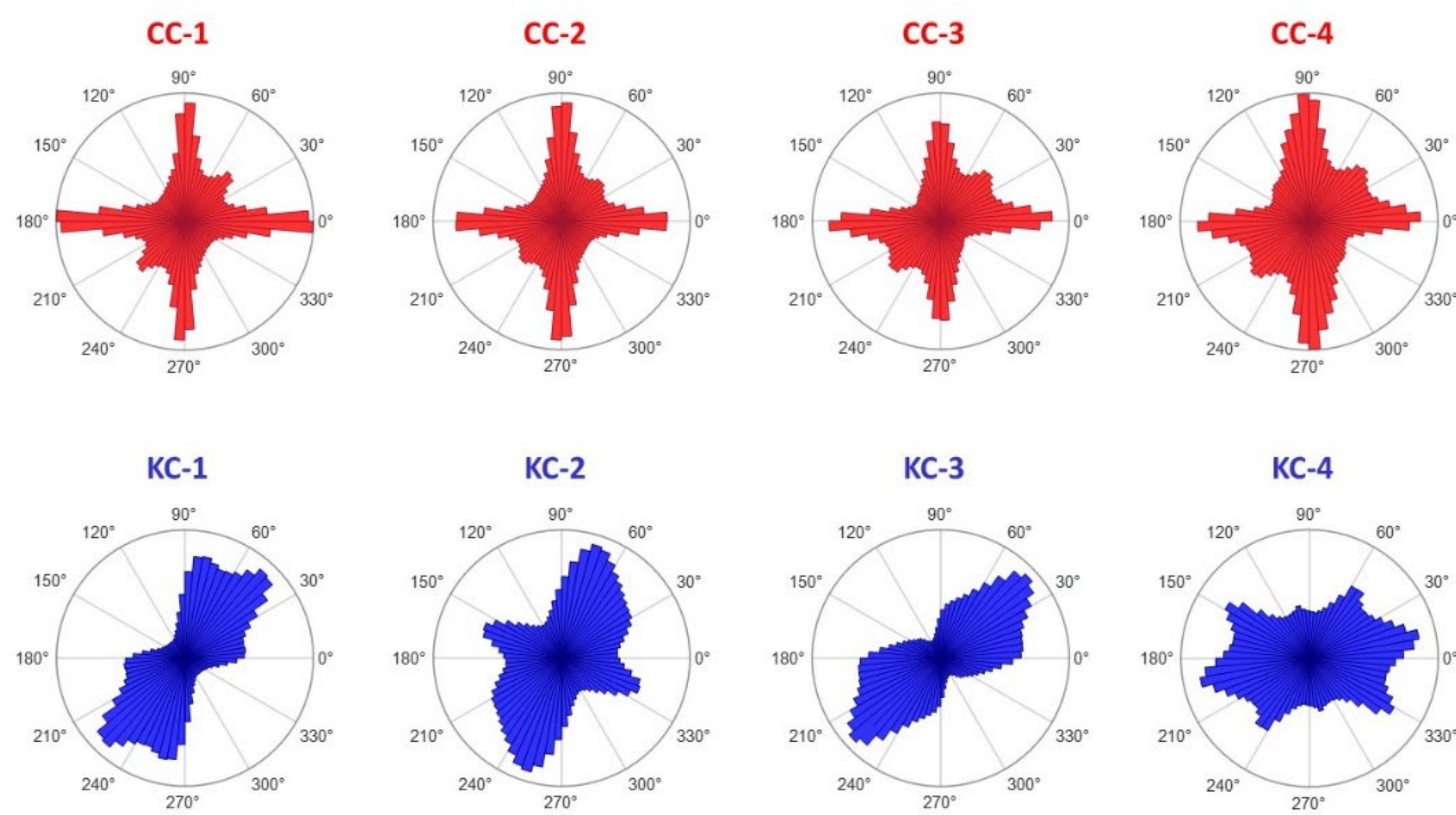

Supplementary Figure S7. Polar plots of voxels orientations obtained from one Z-stack for each cornea using OrientationJ

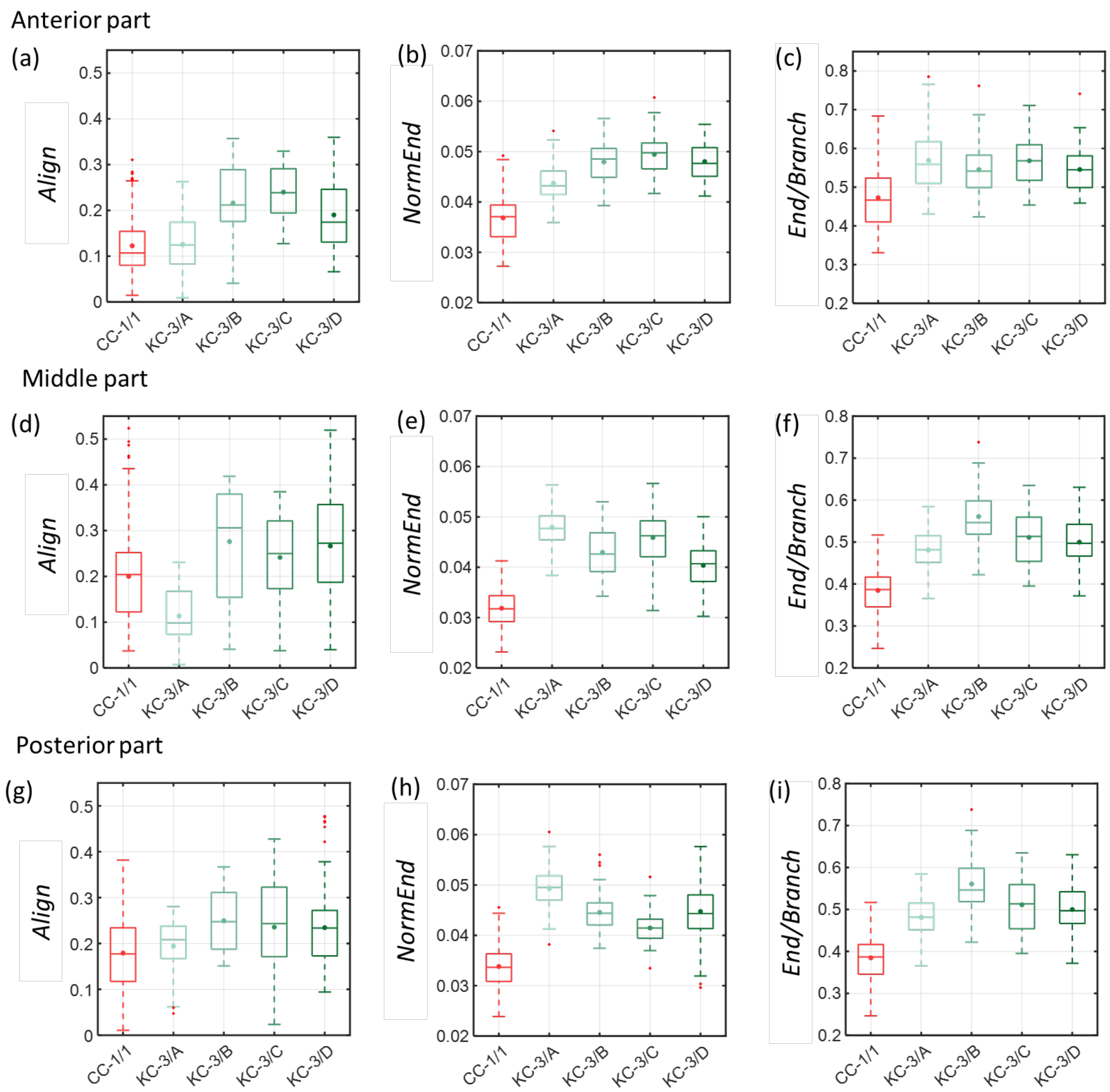

Supplementary Figure S8. Quantitation of SHG striated patterns in the anterior (top), middle (center) and posterior (bottom) parts of the keratoconic cornea (KC-3) as a function of the distance to the cone. Region C is the conical region, regions B and D are located 0.5 mm away from the cone and region A is located 2.5 mm from the cone. Median boxplots of (a,d,g) Align metric, (b,e,h) NormEnd metric and (c,f,i) End/Branch metric for a control cornea (CC-1, one Z-stack) and the 4 Z-stacks of the keratoconic cornea (KC-3).

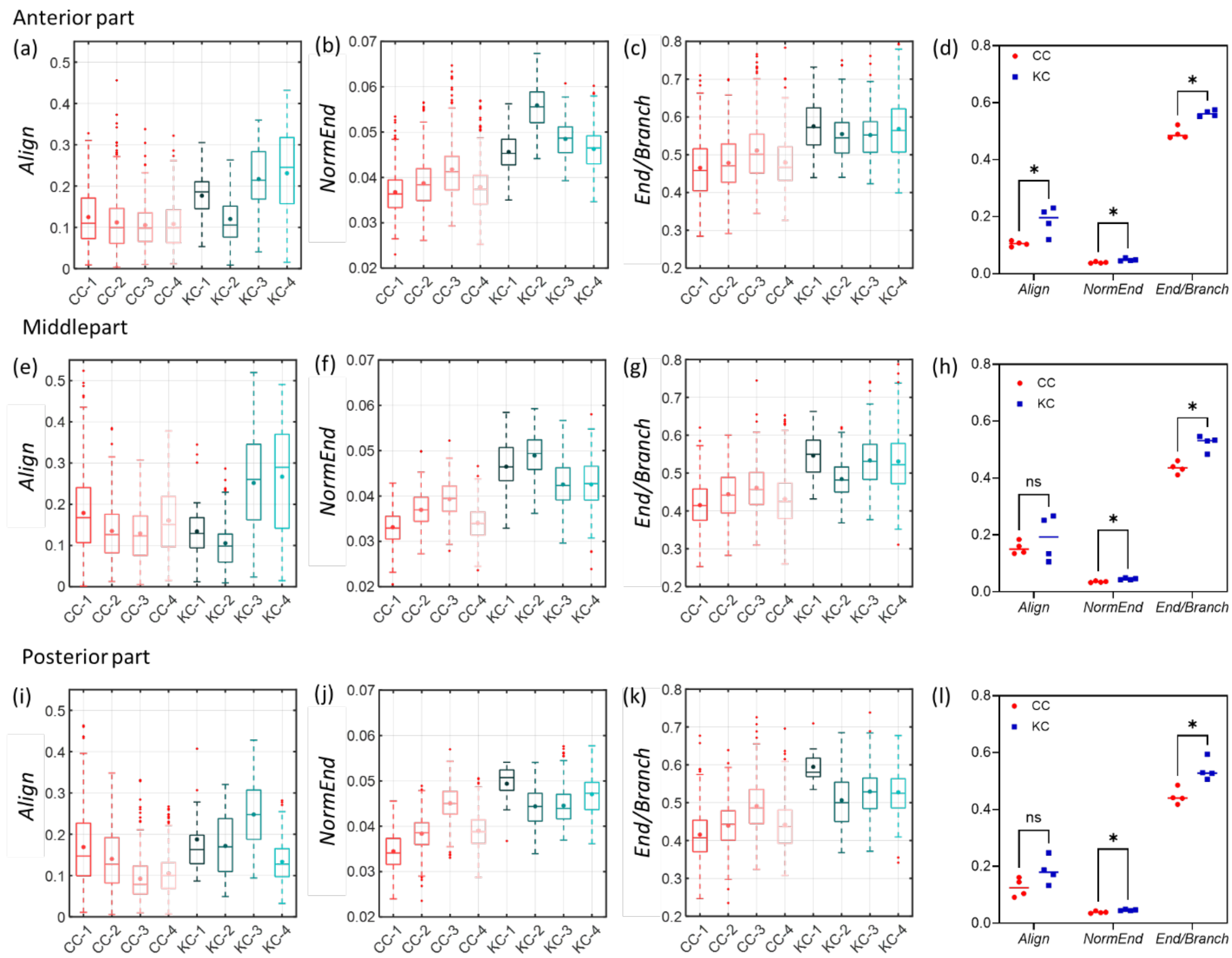

Supplementary Figure S9. Quantitation of SHG striated patterns in the anterior (top), the middle (center) and the posterior (bottom) parts of control (CC) and keratoconic (KC) corneas. (a-c, e-g, i-k) Median boxplots of (a,e, i) Align metric, (b,f,j) NormEnd metric and (c,g,k) End/Branch metric along three Z-stacks for every cornea (except only 2 Z-stacks for KC-1). The mean is plotted as a solid circle. (d,h) Statistical tests based on mean values in each type of cornea.
